# Evolution of clonal hematopoiesis during cancer treatment and its impact on outcomes

**DOI:** 10.1172/JCI204429

**Published:** 2026-06-09

**Authors:** Mona Arabzadeh, Yi-Han Tang, Christelle Colin-Leitzinger, Sadegh Marzban, Daniel Walgenbach, Stefania Morganti, Vaidhyanathan Mahaganapathy, Erika Harper, Mingxiang Teng, Jacob K. Kresovich, Iman Washington, Heather A. Parsons, Judy E. Garber, Jeffrey West, Shridar Ganesan, Hossein Khiabanian, Nancy Gillis

**Affiliations:** 1 Center for Systems and Computational Biology, and; 2 Center for Biomedical Informatics, Bioinformatics Department, Rutgers Cancer Institute, Rutgers University, New Brunswick, New Jersey, USA.; 3 Department of Cancer Epidemiology,; 4 Department of Biostatistics and Bioinformatics, and; 5 Department of Integrated Mathematical Oncology, Moffitt Cancer Center and Research Institute, Tampa, Florida, USA.; 6 Department of Internal Medicine, University of South Florida, Tampa, Florida, USA.; 7 Department of Medical Oncology, Dana-Farber Cancer Institute, Boston, Massachusetts, USA.; 8 Harvard Medical School, Boston, Massachusetts, USA.; 9 Department of Pathology, and; 10 Department of Radiation Oncology, Moffitt Cancer Center and Research Institute, Tampa, Florida, USA.; 11 Department of Medicine, Center for Molecular Oncology, NYU Perlmutter Cancer Center, New York, New York, USA.; 12 Department of Pathology and Laboratory Medicine, Rutgers Robert Wood Johnson Medical School, Rutgers University, New Brunswick, New Jersey, USA.; 13 Department of Malignant Hematology, Moffitt Cancer Center and Research Institute, Tampa, Florida, USA.

**Keywords:** Clinical Research, Genetics, Oncology, Breast cancer, Clonal selection, Hematopoietic stem cells

## Abstract

Clonal hematopoiesis (CH) is the age-related expansion of mutated hematopoietic stem cells without hematologic abnormalities. In patients with solid tumors, CH is associated with higher mortality and may evolve to therapy-related myeloid neoplasms; however, the mechanisms by which cancer treatments promote CH dynamics remain largely unknown. Here, we analyzed 392 serial samples from a prospective cohort of patients with breast cancer and show that cytotoxic treatments led to strong therapeutic bottlenecks, resulting in significant reductions in hematopoietic allelic populations and differential clonal selection. Positively selected CH that expanded through dose-dependent therapeutic bottlenecks harbored mutations in *TP53*, *PPM1D*, *SRCAP*, *DNMT3A*, and *YLPM1*. Patients with positively selected CH during treatment had the shortest progression-free and overall survival compared with patients with unchanging or negatively selected CH across all therapies. These findings, validated in independent breast cancer and pan-cancer cohorts, provide strong evidence for the clinical relevance of monitoring CH during cancer treatment.

## Abstract 2

Cancer treatment creates positive selection pressure, reducing allelic diversity while promoting expansion of specific mutant clones associated with clinical outcomes.

## Graphical abstract

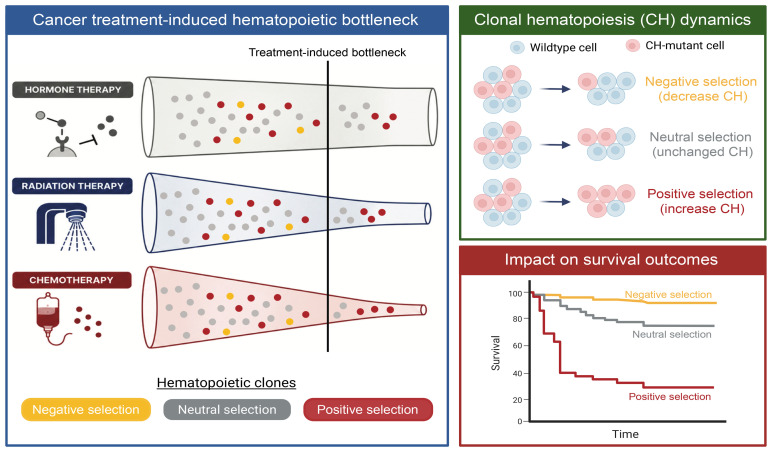

## Introduction

Clonal hematopoiesis (CH) is caused by the expansion of hematopoietic stem cells that carry somatic alterations in genes recurrently mutated in myeloid neoplasms. CH mutations with variant allele frequencies (VAFs) of 2% or higher, defined as clonal hematopoiesis of indeterminate potential (CHIP), are associated with increased overall mortality, cardiovascular disease, and progression to hematologic neoplasms (1–4). CH mutations are more common in individuals with solid tumors compared with healthy population-based cohorts (5) and are routinely detected in blood and tumor specimens (6–8).

The growth pattern and Darwinian evolution of mutation-driven CH resemble cancer and are shaped by gene-specific fitness effects, hematopoietic cell–specific rates of mutation, and imposed adaptive pressure on hematopoiesis (9–14). Cross-sectional studies have demonstrated a higher prevalence of CH mutations, particularly at CHIP-defining levels, following exposure to cancer therapy (15). Longitudinal studies in cancer-free populations and patients with solid tumors have further shown mutation-specific CH evolution and progression to myeloid malignancies (16–20). Analyses in patients with solid tumors reveal distinct mutational patterns and clinical associations under cancer treatment (21–25); however, the evolutionary mechanisms driving differential CH clonal dynamics and their relationship to clinical outcomes are largely unknown.

Extrinsic selection pressures imposed by cancer therapy may induce hematopoietic stress that promotes CH clonal expansion or leukemic transformation or, conversely, lead to depletion of mutated hematopoietic cell populations after treatment for solid tumors (26–28). Restriction of therapeutic bottlenecks results in deep reductions in hematopoietic allelic populations, establishing an evolutionary setting in which fitter clones preferentially expand, while less fit or neutral clones may or may not persist due to highly stochastic random drift.

To investigate the impact of cancer treatment on mutation-driven CH, we analyzed 392 serial peripheral blood samples collected from patients with breast cancer before, during, and after treatment. We quantified treatment-specific selective pressures, clonal fitness, and effective hematopoietic cell population sizes, and evaluated associations between CH dynamics and clinical outcomes (Supplemental Figure 1). Our findings were validated in independent cohorts of patients with breast and pan-cancer tumors.

## Results

### CH mutational spectrum at breast cancer diagnosis

**Figure 1:**
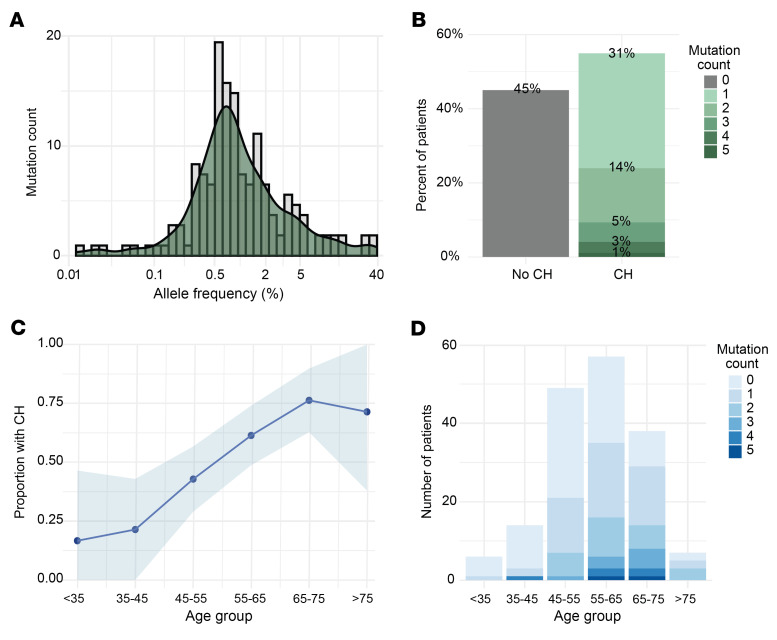
Prevalence and mutational distribution of CH prior to treatment for breast cancer. (**A**) Density distribution of CH mutation VAFs. (**B**) Percentage of patients with or without CH, stratified according to the number of detected mutations per individual. (**C**) Proportion of patients with CH across age groups. (**D**) Number of patients with CH across age groups, stratified on the basis of the number of detected mutations per individual.

Mapping the mutational landscape of CH mutations in samples collected before treatment initiation (range, 1 day to 7.7 months) revealed 160 protein-changing CH mutations (VAF ≥0.01%) in 55% of patients (94 of 171) (Figure 1, A and B), across the genes included in our targeted sequencing panel (Supplemental Table 1). Patients with CH at diagnosis were older than those without CH (median age, 62 vs. 52 years; *P* < 0.001) (Table 1). The prevalence of CH increased with age, reaching 75% among patients aged 65 to 75 years (Figure 1C). The median number of CH mutations within an individual was 1 (range, 0–5) and was associated with age (*P* = 0.04) (Figure 1D).

**Figure 2:**
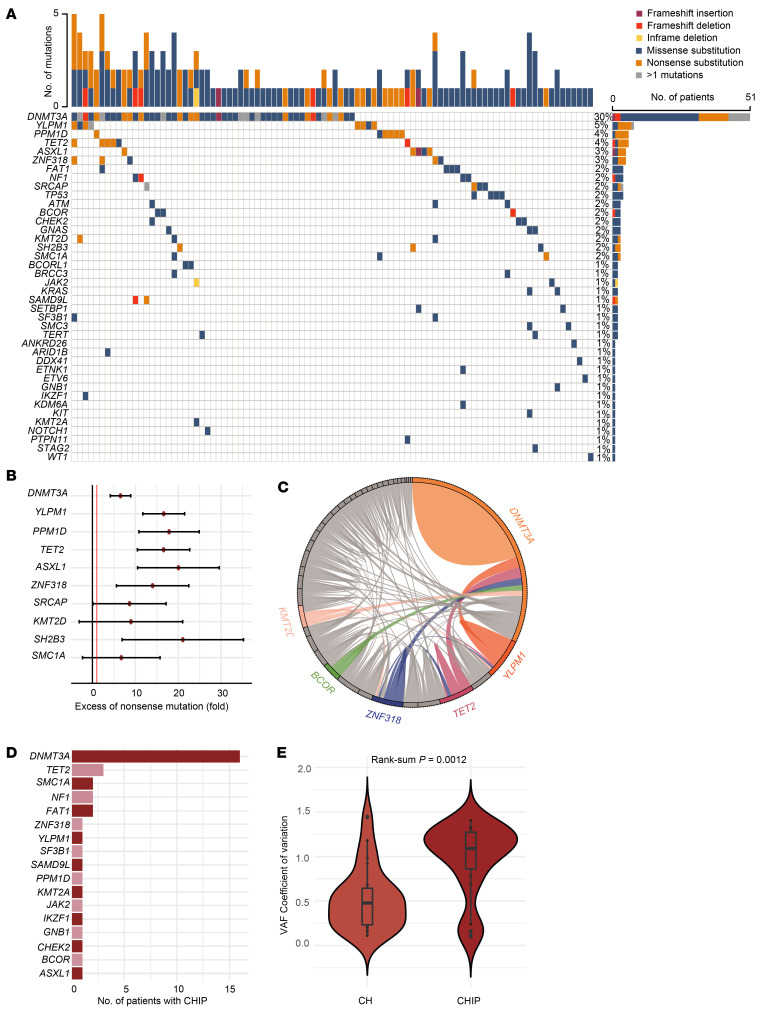
Mutational spectrum and diversity of CH prior to treatment for breast cancer. (**A**) Oncoplot of CH mutations detected across a panel of 81 genes in patients with breast cancer. Columns represent individual patients, and colors correspond to the mutation type, including frameshift insertion and deletions, in-frame deletions, and missense and nonsense single nucleotide variants. (**B**) Fold excess of nonsense mutations in each gene relative to the expected distribution from the genes’ nucleotide content; χ^2^ statistics are shown with 95% CIs for genes with 3 or more protein-changing substitutions and 1 or more nonsense mutations. (**C**) CH mutation co-occurrence illustrated by a Circos plot. Colored lines highlight the most frequently mutated genes and co-mutations. (**D**) Number of patients with CHIP mutations (VAF ≥2%) across genes. (**E**) Divergence in CH allelic frequency, defined by the mean CH VAF per patient, compared between patients with CHIP mutations (VAF ≥2%) versus patients with CH mutations (VAF <2%).

In *DNMT3A*, *TET2*, and *ASXL1*, the most frequently mutated CH genes in healthy populations (29, 30), we detected missense, loss-of-function nonsense, and frameshift mutations in 60% of patients with CH mutations (60 of 94) (Figure 2A and Supplemental Table 2; supplemental material available online with this article; https://doi.org/10.1172/JCI204429DS1). To assess evidence of clonal selection (31), we compared observed mutation classes with expectations based on nucleotide composition. In 83% of genes with at least 5 nonsynonymous mutations (5 of 6 genes), nonsense substitutions were enriched 7- to 18-fold relative to neutral expectations (FDR <0.001) (Figure 2B). Although the limited number of missense substitutions in the remaining genes did not provide power to assess their clonal selection (Supplemental Table 3), these results corroborate the extent of population-level, positive selection of loss-of-function mutations in CH (9).

### CH mutational diversity at breast cancer diagnosis

The most frequent co-occurring CH mutations involved *DNMT3A*, which was found to be co-mutated with *TET2* (*n* = 5; OR = 4.50, *P* = 0.14), *YLPM1* (*n* = 4; OR = 0.83; *P* = 0.73), and *ZNF318* (*n* = 3; OR = 1.20; *P* = 0.57) (Figure 2C), although this did not reach statistical significance. At least 1 CHIP mutation (strictly defined here by a VAF ≥2%) was detected before treatment in 33% of patients with CH (31 of 94), most commonly in *DNMT3A* (*n* = 15) and *TET2* (*n* = 3) (Figure 2D). Among patients with more than 1 CH mutation (*n* = 41), clonal diversity measured by the coefficient of variation of VAFs had a median of 0.68 (range, 0.10–1.44) and was significantly higher in patients with at least 1 CHIP mutation compared with those without CHIP-level mutations (rank-sum *P* = 0.001, Figure 2E), even after excluding mutations with a VAF of 2% or higher from the analysis (rank-sum *P* = 0.02, Supplemental Figure 3A). These results underscore the notion that as early CH clones grow in abundance, a larger number of CH mutations may accumulate and give rise to increased clonal diversity.

### CH mutational rise and fall during breast cancer treatment

**Figure 3:**
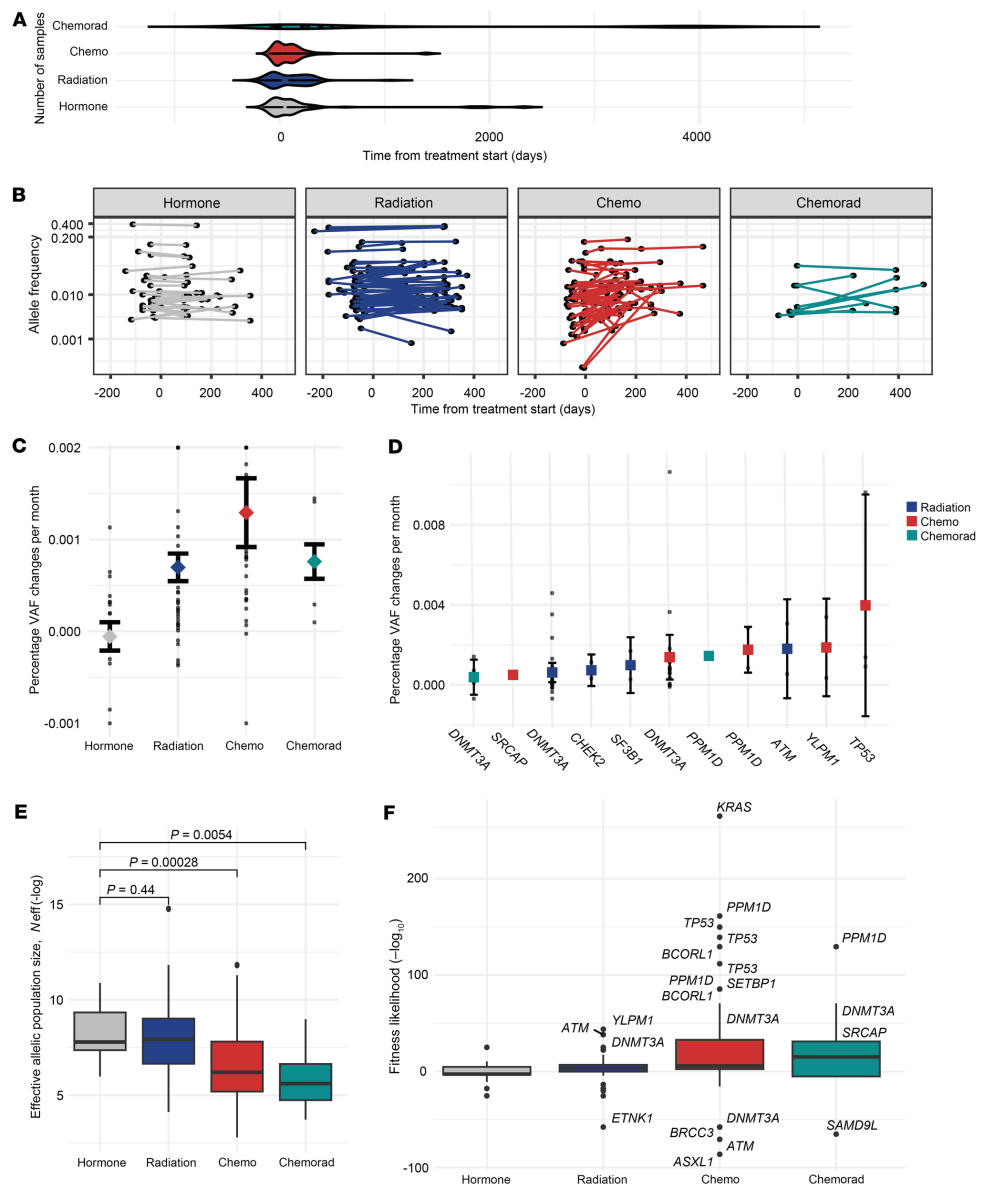
CH mutational dynamics during breast cancer treatment. (**A**) Number and temporal spread of longitudinal samples collected by treatment modality; times are presented relative to treatment start (time = 0). (**B**) Measured VAF for all detected CH mutations during the study by treatment modality. (**C**) Standardized percentage change in VAF per month for all detected mutations by treatment modality. (**D**) Standardized percentage change in VAF per month for gene-specific mutations showing a substantial allelic increase per treatment modality. (**E**) Effective allelic population size (*N*_eff_) across treatment modalities (rank-sum test). (**F**) Fitness likelihood, defined by the minus log of probability of observing post-treatment VAF of a CH mutation given the measured effective allelic population size per patient, across treatment modalities; genes with mutations showing the highest CH clonal change relative to the WT population are indicated. chemo, chemotherapy; chemorad, chemo-radiation.

To evaluate clonal dynamics under treatment, we analyzed 230 serial peripheral blood samples (*n* = 1–4 per patient) collected at a median of 5.5 months after treatment initiation (range, 0.01–132.3 months) (Figure 3A and Supplemental Figure 2). All CH mutations detected after treatment initiation were present in pretreatment samples, enabling longitudinal tracking of VAF changes (Figure 3B).

#### Short-term impact of treatment on CH allele frequency.

Within the first 18 months of treatment initiation, the maximum normalized monthly change in VAF was highest among patients receiving chemotherapy (0.13% ± 0.04% per month), followed by combination chemotherapy and radiation (0.08% ± 0.02% per month) and radiation alone (0.07% ± 0.02% per month) (Figure 3C and Supplemental Table 4). In contrast, patients receiving hormonal therapy alone showed no measurable change in CH VAFs during this period (–0.005% ± 0.02% per month).

The fitness landscape of CH is gene and mutation dependent (9, 12, 14). Gene-specific analyses showed that among patients treated with chemotherapy alone, *TP53*-CH exhibited the greatest average standardized VAF increase (0.40% ± 0.28% per month), followed by *YLPM1* mutations (0.19% ± 0.12% per month) (Figure 3D and Supplemental Table 4). Radiation therapy was associated with measurable VAF increases in *ATM*-mutated clones (0.18% ± 0.13% per month). No gene-specific VAF changes were observed among patients receiving hormone therapy alone, and *DNMT3A* mutations showed significantly smaller VAF changes compared with other treatment modalities (rank-sum *P* < 0.02) (Supplemental Figure 3B). All CHIP-level mutations detected before treatment remained at CHIP-level VAFs after treatment (Supplemental Figure 3C).

#### Long-term impact of treatment on CH allelic abundance.

To assess long-term effects, we analyzed 10 samples collected from 6 patients 22–133 months after treatment initiation. CH mutations showed continued changes in VAFs over time, with the largest increases observed among patients treated with cytotoxic chemotherapy. In 2 patients, *DNMT3A* mutations increased by more than 0.1% VAF per year across 4–8 years of follow-up (Supplemental Table 4).

### CH evolutionary dynamics during breast cancer treatment

#### Effective allelic population size during treatment.

To quantify selective pressures during treatment, we estimated the effective allelic population size (*N_eff_*) using pre- and post-treatment VAFs, adapting the method for estimating pathogen transmission population size (32–34). We interpreted *N_eff_* as an estimate that captures the magnitude of stochastic drift in mutated hematopoietic cell populations during treatment, rather than as the absolute number of hematopoietic stem or progenitor cells. As such, *N_eff_* reflects the relative size of the therapeutic bottleneck acting on the evolving hematopoietic compartment, where smaller values indicate stronger drift and greater clonal fluctuation.

In our cohort during the first 18 months of treatment, median *N_eff_* was the highest among patients receiving hormonal therapy (median, 3,804 alleles; range, 397–53,206) and radiation (median, 2,765; range, 62–1,000,000). In contrast, *N_eff_* was significantly lower among patients receiving chemotherapy (median, 496; range, 16–135,888), similar to combination chemotherapy and radiation (median, 277; range, 42–798,800), pointing to substantially more restrictive therapeutic bottlenecks imposed by cytotoxic treatment of breast cancer (Figure 3E).

### CH mutational fitness during treatment

Using patient-specific effective allelic population size (*N_eff_*) estimates and longitudinal VAF measurements, we quantified evolutionary fitness of CH mutations relative to the WT alleles during treatment. Specifically, we evaluated whether the observed change in VAF after treatment could be explained by neutral genetic drift alone. For each mutation, we calculated the probability of observing the post-treatment VAF given its pretreatment VAF, assuming a neutral sampling process from an allelic population size of *N_eff_*, and classified the CH mutations as positive or negative selection only when the observed VAF change was unlikely to arise from drift alone. Mutations showing significant expansion beyond drift expectations, with fitness likelihoods exceeding 10,000-fold (>5 orders of magnitude) relative to WT alleles, were classified as positively or negatively selected according to the direction of the VAF change, whereas mutations whose VAF changes were consistent with drift were classified as under no selection.

In our cohort, within the first 18 months of the start of treatment, the highest mutational fitness was observed under chemotherapy. Among patients treated with chemotherapy or combination therapy, 27% (22 of 81) had positively selected mutated clones (Supplemental Table 5). Under radiation, *DNMT3A* mutations showed the highest relative fitness (Figure 3F). The fittest *PPM1D*-mutant clones had truncating mutations in exon 6 that were linked to a chemoresistance phenotype (35), and positively selected *DNMT3A* mutations were in the PWWP/chromatin-binding (exons 8–9) and DNA methylase (exons 16–19) domains (Supplemental Figure 4).

#### CH mutational dynamics across treatment modalities and dosage levels.

CH has been shown to differentially respond to cancer treatment based on the mechanism of action and dosage. Among patients with pretreatment CH, 38% (36 of 94) exhibited dynamic VAF changes within the first 18 months, including 9% (3 of 35) receiving hormonal therapy, 21% (7 of 55) radiation, 30% (21 of 69) chemotherapy, and 42% (5 of 12) combination therapy. In 3% of patients (3 of 94), all treated with radiation, both increases and decreases in CH VAFs were observed.

**Figure 4:**
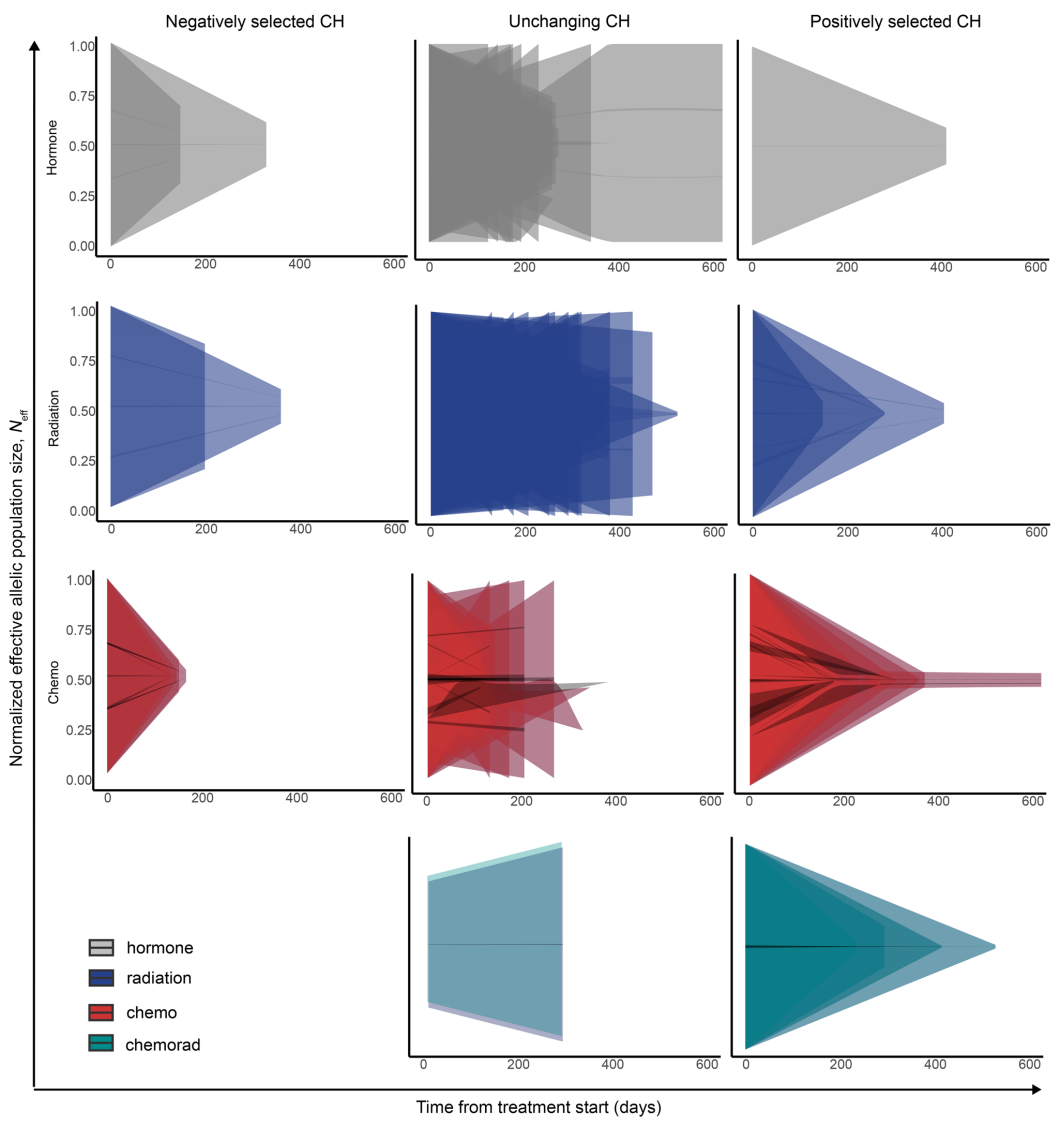
Allelic population bottlenecks imposed by cancer treatment. Schematics showing effective allelic population size (*N*_eff_) across treatment modalities, normalized by mean *N*_eff_ in patients treated with hormonal therapy only.

**Figure 5:**
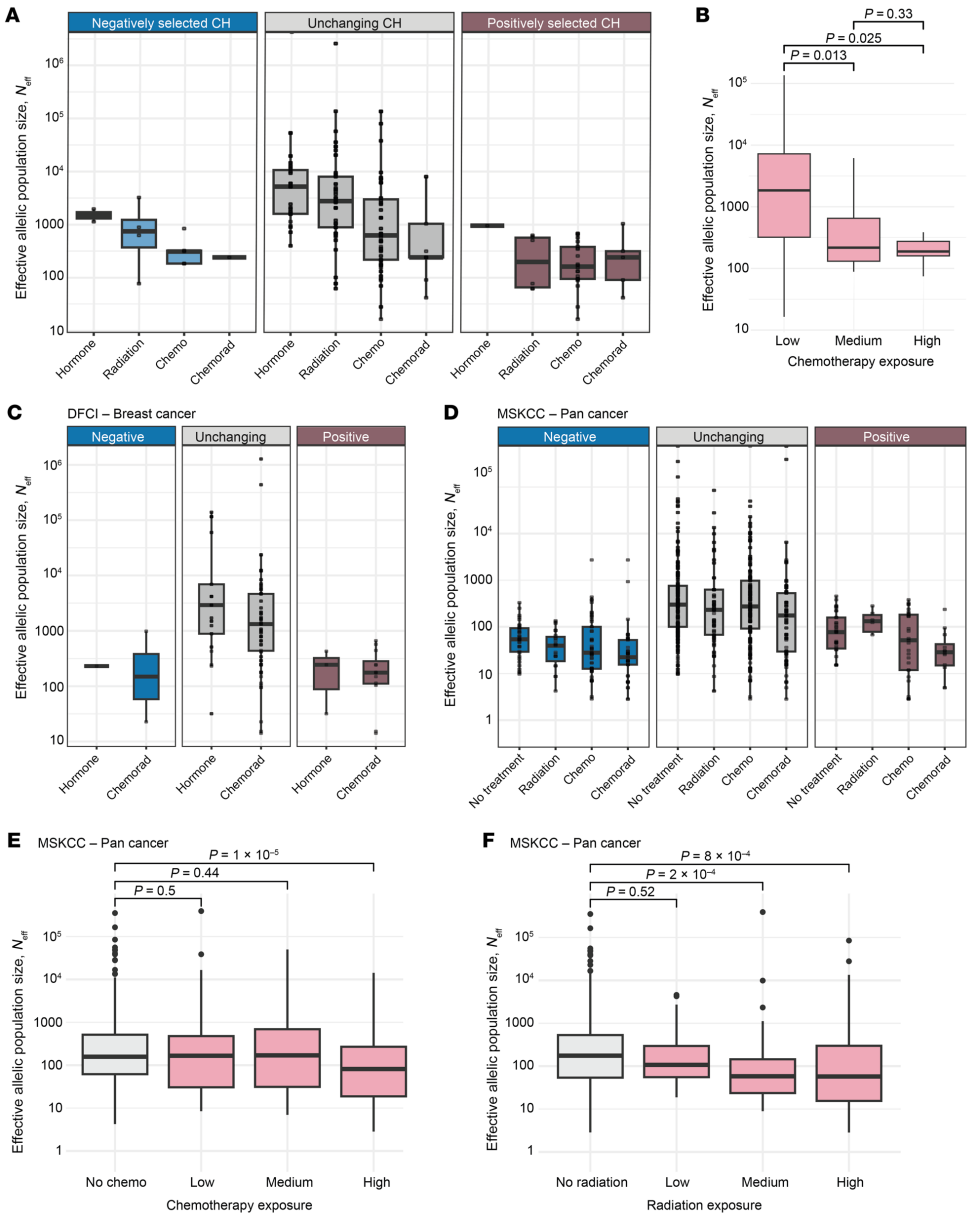
Effective allelic populations associate with treatment modality and dosage. (**A**) Effective allelic population sizes (*N*_eff_) by treatment modalities, grouped on the basis of CH mutational dynamics, including positively selected, unchanging, and negatively selected CH. (**B**) *N*_eff_ distributions across cumulative cytotoxic chemotherapy exposure levels (rank-sum test). (**C**) *N*_eff_ in patients with breast cancer treated at the DFCI, by treatment modalities, and grouped according to CH mutational dynamics, including positively selected, unchanging, and negatively selected CH. (**D**) *N*_eff_ in patients with solid tumors treated at the MSKCC, by treatment modalities, and grouped on the basis of CH mutational dynamics, including positively selected, unchanging, and negatively selected CH. (**E**) *N*_eff_ distributions across cumulative chemotherapy exposure levels in patients with solid tumors treated at the MSKCC (rank-sum test). (**F**) *N*_eff_ distributions across cumulative radiotherapy exposure levels in patients with solid tumors treated at the MSKCC (rank-sum test).

Effective allelic population sizes were smaller among patients with positively or negatively selected CH compared with those with stable CH, with the most pronounced contractions observed among patients receiving chemotherapy or combination therapy, suggesting that therapeutic elimination of WT cells may be a major driving factor behind the observed evolutionary fitness of selected CH mutations (Figure 4, Figure 5A, and Supplemental Figure 5).

Patients with or without CH had similar levels of chemotherapy exposure. Among patients with CH who were treated with chemotherapy (*n* = 36), increasing cumulative cytotoxic exposure was associated with higher frequencies of CH dynamics (χ^2^ *P* = 0.003). In addition, patients with high or medium cumulative exposure had significantly smaller *N_eff_* during cytotoxic therapy relative to patients with low-dosage treatment (rank-sum *P* = 0.025 and *P* = 0.013, respectively; Figure 5B). No significant association was observed between radiation dosage and *N_eff_*. In fact, it is the dose-dependent severity of bottlenecks imposed by systemic cytotoxic therapy that yields shrinking hematopoietic cell populations which, together with mutation-specific resistance of CH clones to chemotherapeutic drugs (36), may result in increased CH VAFs during treatment.

### CH mutational dynamics validation across independent cancer cohorts

Analysis of independent cohorts from the Dana-Farber Cancer Institute (DFCI) (early-stage breast cancer, *n* = 62) (22) and Memorial Sloan Kettering Cancer Center (MSKCC) (solid tumors, *n* = 394) (5) confirmed smaller effective allelic populations and stronger selective pressures among patients treated with cytotoxic therapies compared with those receiving hormonal therapy or no cytotoxic treatment (Figure 5, C and D, and Supplemental Figures 6 and 7). Moreover, there was a consistent relationship between cytotoxic exposure levels and CH dynamics across all cancers (MSKCC cohort), where positive or negative selection of mutations was significantly associated with higher chemotherapy (χ^2^ *P* = 0.001) or radiation dosage (χ^2^ *P* = 1 × 10^–4^) (Supplemental Figure 7). *N_eff_* sizes were significantly smaller in patients who received the highest cumulative dosage of chemotherapy (rank-sum *P* = 1 × 10^–5^, Figure 5E) or were exposed to high or medium levels of radiation compared with no cytotoxic treatment (rank-sum *P* = 2 × 10^–4^ and *P* = 8 × 10^–4^, respectively, Figure 5F). These results confirm the relationship between cytotoxic treatment dosage and quantified reduction in hematopoietic cell populations (37), pointing to CH clonal dynamics as a biomarker for outcomes across cancers.

### Association of CH dynamics with clinical outcomes

**Figure 6:**
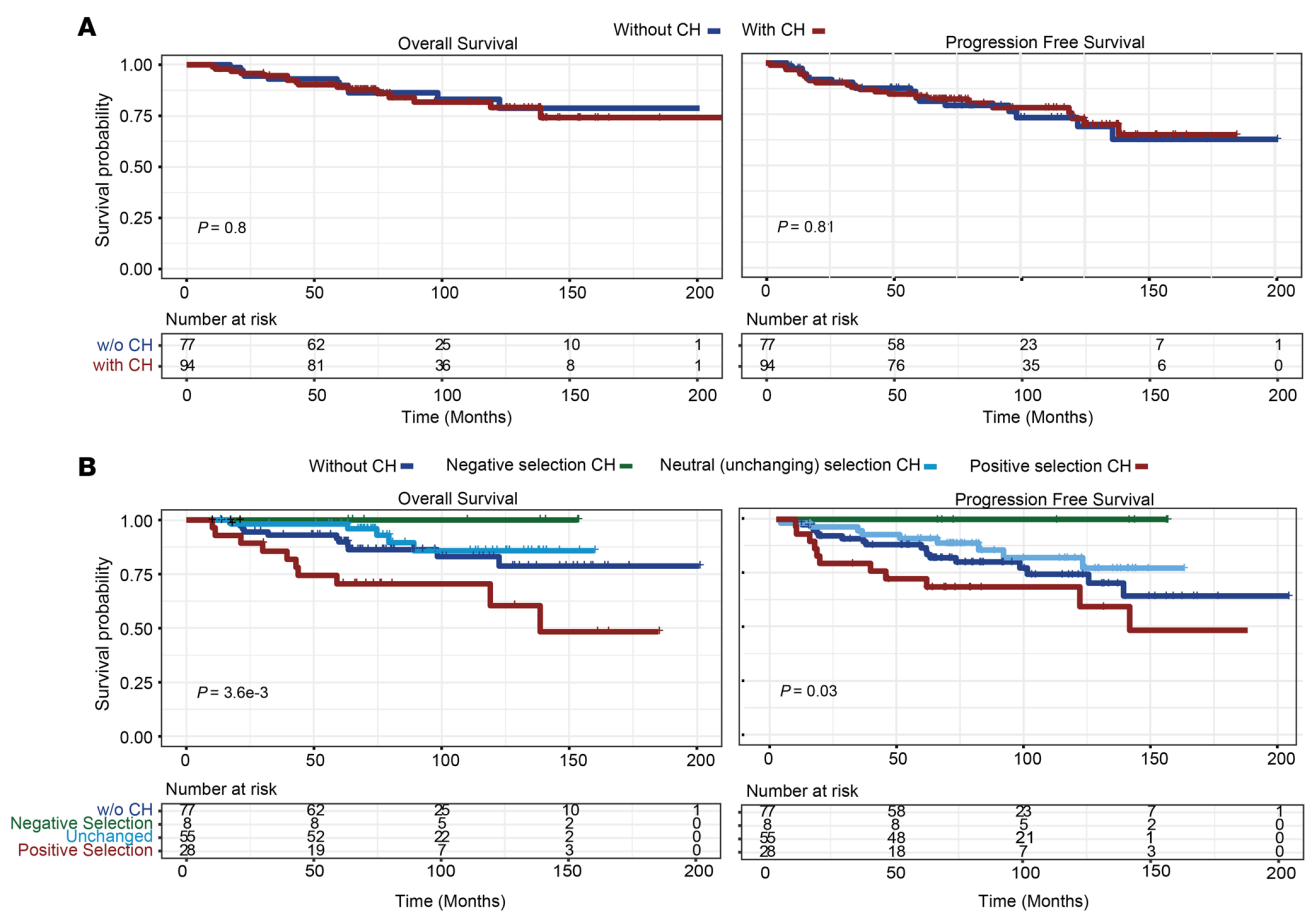
CH mutational dynamics and patient survival. (**A**) Difference in OS and PFS between patients with and without CH (log-rank test). (**B**) Differences in OS and PFS between patients with positively selected, negatively selected, unchanging, and no CH. Statistical significance was determined by log-rank test.

**Figure 7:**
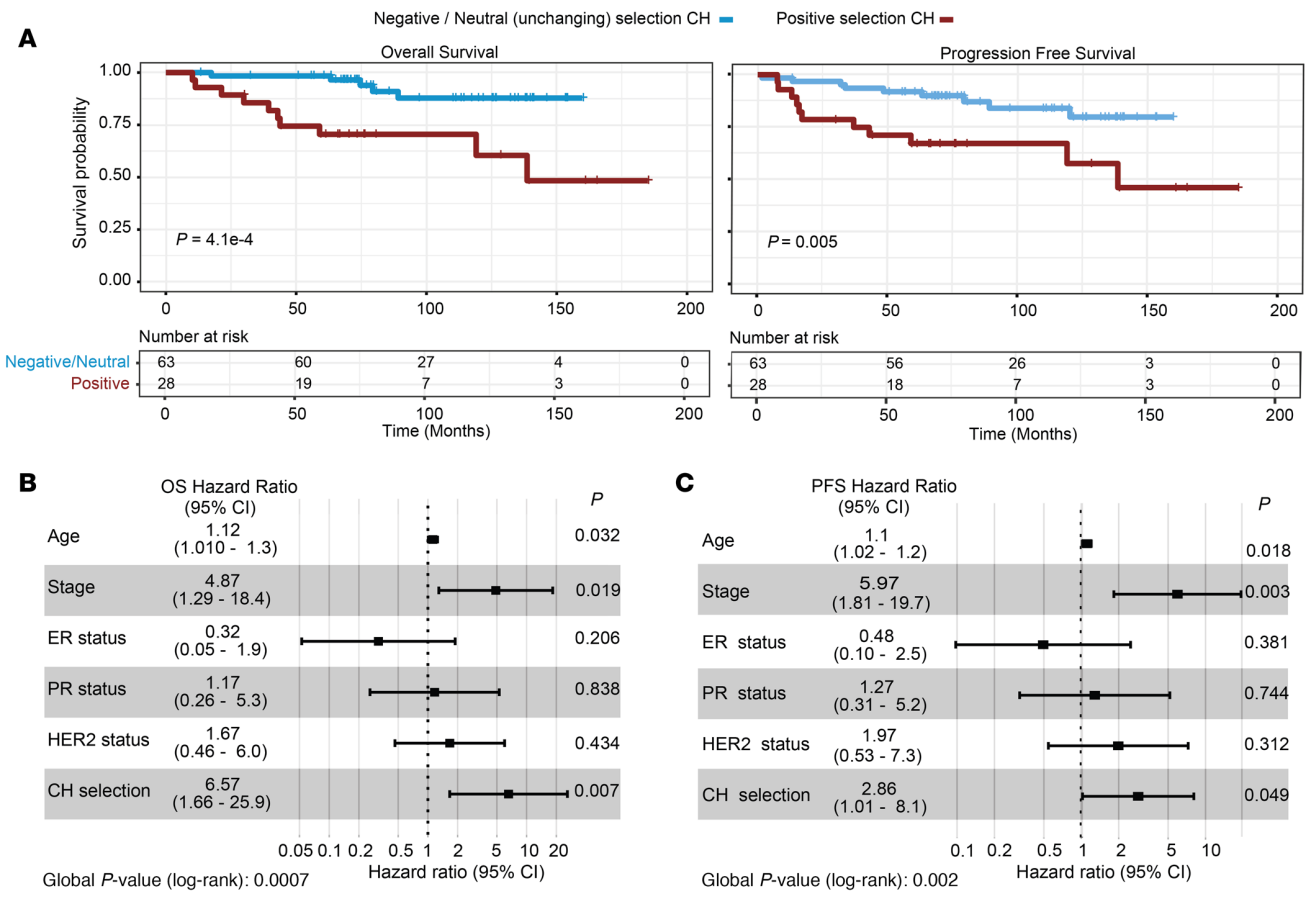
Positive selection of CH affects patient survival. (**A**) Differences in OS and PFS between patients with positively selected CH and those with negatively selected or unchanging CH (log-rank test). (**B**) The effect of positively selected CH on OS using a multivariable Cox regression model including patient age, tumor stage and ER, PR, and HER2 hormonal status. (**C**) The effect of positively selected CH on PFS using a multivariable Cox regression model including patient age, tumor stage and ER, PR, and HER2 hormonal status. Kaplan-Meier plots with log-rank *P* statistics are shown.

Overall survival (OS) and progression-free survival (PFS) did not differ between patients with or without CH before treatment (Figure 6A). However, among patients with CH, those with positively selected mutations during treatment had significantly worse OS and PFS (Figure 6B and Figure 7A). In multivariable Cox regression models adjusted for age, stage, and estrogen receptor (ER), progesterone receptor (PR), and human epidermal growth factor receptor 2 (HER2) status, positive CH selection was associated with shorter OS (adjusted hazard ratio, 6.6; 95% CI, 1.7–25.9; *P* = 0.007) and worse PFS (adjusted hazard ratio, 2.86; 95% CI, 1.01–8.1; *P* = 0.049) (Figure 7, B and C, and Supplemental Table 6). These results were further supported by landmark analyses at 12 and 18 months as well as by a time-dependent Cox model incorporating patient-specific sample collection time points, all of which yielded results consistent with multivariable and univariable Cox models (Supplemental Table 6).

These associations persisted in analyses restricted to patients receiving chemotherapy and in recurrence-free survival analyses (Supplemental Figure 8). When considering all patients who received chemotherapy, there was no association between cumulative chemotherapy dose and OS; however, multivariable analyses assessing the relationship between chemotherapy exposure level and CH dynamics were limited by small samples within the subgroups (Supplemental Table 6). Among patients who received similar treatment regimens, positively selected patients with CH tended to have worse outcomes relative to negatively selected or unchanged patients with CH, although statistical significance was not reached for these subgroup analyses (Supplemental Figure 9). Together, these results link CH clonal dynamics during treatment to patient mortality and disease outcome and, for the first time to our knowledge, provide quantified evidence for the observed association between mutation-driven CH and OS (31).

## Discussion

In this longitudinal analysis of patients with breast cancer, we demonstrate that CH was present in 55% of patients, evolved dynamically during treatment, and was strongly shaped by therapy-specific selective pressures. By integrating high-sensitivity sequencing (detect >1 mutant in 1,000 WT alleles [ref. 10]) with evolutionary modeling, we show that cytotoxic therapies imposed pronounced therapeutic bottlenecks on hematopoietic cell populations, leading to clonal expansion of fitter CH mutations. Importantly, we identify treatment-associated CH dynamics, rather than CH presence alone, as a predictor of adverse clinical outcomes. All post-treatment CH mutations were detectable at baseline, supporting the notion that cancer therapy selectively modulated preexisting CH rather than initiating the development of de novo CH (26).

The fitness landscape of CH is shaped by multifactorial processes such as aging, inflammation, germline predisposition (38, 39), and DNA-damaging exposures (31, 40). Mutations in the DNA damage response genes, including *TP53* and *PPM1D*, have been implicated in therapy-related myeloid neoplasms (5, 15, 40, 41). *SRCAP* and *YLPM1* have also been recognized as drivers of CH and myelodysplastic neoplasms (9, 42), with *SRCAP* linked to selective advantage under cytotoxic stress (43). Here, *YLPM1* emerged as a CH driver that persisted and expanded under therapy, suggesting a potential role in hematopoietic stress. Among common CH drivers, *DNMT3A* mutations selectively expanded under both chemotherapy and radiation. *DNMT3A* has been linked to chemotherapy resistance in hematologic malignancies (44) and associated with comorbidities in solid tumors (45, 46). Here, we found that *DNMT3A* mutations frequently co-occurred with *TET2*, *YLPM1*, and *ZNF318*, suggesting potential clonal cooperation or cosegregation; however, *TET2* mutations in blood did not show evidence of dynamic selection under therapy. Given that *TET2*-mutant CH is enriched in the tumor microenvironment and associated with tumor progression (7, 8), a possible explanation may be that *TET2* acts as a context-dependent co-driver by remodeling the tumor immune microenvironment, rather than via a direct proliferative advantage. This indirect role may support the fitness of *TET2*-mutant clones even without overt selection under therapy.

To assess whether the observed differences reflect biological selection between treatment groups, we examined VAF changes for *DNMT3A*, *YLPM1*, *TP53*, and *ATM* separately among chemotherapy- and radiation-treated cohorts (Supplemental Figure 3). The observed patterns were consistent with differential selective pressures (chemotherapy favoring *DNMT3A*, *TP53*, and *YLPM1*, and radiation favoring *ATM*) but should be considered hypothesis generating rather than definitive, given the limited statistical power. We observed evidence suggestive of positive selection of *TP53* mutations in chemotherapy-treated patients, consistent with prior reports linking *TP53*-mutant hematopoietic clones to resistance to DNA damage–induced apoptosis (36). In contrast, no clear signal of *TP53* selection was observed in the radiation-treated group. In addition, the enrichment of *ATM* mutations in the radiation-treated group is biologically plausible, given the central role of *ATM* in the DNA damage response to double-stranded breaks and radiation-induced genotoxic stress (47), supporting the possibility of treatment-specific selective pressure.

We independently confirmed the associations between CH dynamics, reduction in effective allelic population size, and cumulative cytotoxic exposure in other cohorts, denoting that genotoxic stress promotes relative expansion of clones with higher intrinsic fitness. This enrichment aligns with known links between CH and therapy-related neoplasms and may reflect therapy-specific sensitivity or immune-mediated clearance of less fit clones. The reduced impact of radiation on *N_eff_* and CH selection may reflect the limited hematopoietic effect of breast-directed radiation and may not generalize to tumor types where radiation treatment more directly affects large regions of the hematopoietic stem cell compartment (48) (e.g., pelvic radiation for gynecologic cancers [ref. 49]). Consistent with these findings, our pan-cancer analysis supports the likelihood for dose-dependent, radiation-induced CH positive selection in other cancer types.

CH evolution and growth have been linked to cancer therapy–related adverse sequelae (5, 15, 18, 20). Although none of the patients in our cohort developed therapy-related hematological malignancies during the study period, stratification by CH clonal trajectories showed that patients with positively selected CH had an increased risk of mortality compared with those with neutral or negatively selected CH, suggesting that the direction of clonal evolution, rather than CH presence, is prognostically meaningful. This effect persisted when the analysis was limited to only patients who underwent chemotherapy and to groups with homogenous treatment.

The mechanism linking positive selection of CH clones to worse outcomes remains unclear. In our analyses, CH dynamics were primarily detected in genes with mutations linked to therapeutic resistance, and their association with poor outcomes might therefore be confounded by the extent of treatment exposure due to progressive disease. Although no overall association was observed between chemotherapy dose and outcomes across our whole cohort, exploratory analyses suggested a differential relationship based on CH selection status. In patients with negative selection of CH, the chemotherapy dose had no apparent effect on outcomes, however, in positive selection, higher cumulative doses may have been associated with improved outcomes. These observations further suggest that CH selection status may serve as an independent predictor of treatment response and that the positive selection of CH is influenced by factors beyond the chemotherapy dose alone. In support of the direct role played by tumor-infiltrating CH clones in tumor growth and treatment response (8, 50, 51), the association between positive selection for CH clones in peripheral blood and outcomes also raises the possibility that circulating CH clones have some direct or indirect effects on treatment response.

Our study provides a distinct advance over previous large retrospective CH analyses by shifting from a static, cross-sectional framework to a prospective, longitudinal evaluation of clonal hematopoiesis dynamics during cancer treatment. Although our cohort is smaller than those in earlier retrospective studies (5, 15), the design of our study enabled direct measurement of how CH clones evolve under toxic therapy, supported by a hormone therapy control group, and showed that the observed changes reflected both natural time-dependent drift and treatment-specific selective pressures. High-depth sequencing enhanced analytic resolution by detecting subtle shifts in clonal architecture that would be missed at lower coverage.

Despite the modest sample size, our analyses within more homogeneous treatment groups revealed consistent patterns across CH dynamic–stratified groups, reinforcing the robustness of these observations. By tracking clonal trajectories rather than cataloging the presence of CH, we demonstrate that therapy-driven clonal expansion was associated with earlier tumor progression — an association not captured by prior work focused predominantly on enrichment patterns and leukemia risk. We further show that therapy-dependent reductions in hematopoietic allelic population size shaped clonal fitness across treatment modalities. Notably, CH selection may not necessarily represent a causal driver of adverse outcomes but may instead reflect underlying physiological vulnerability or conditions promoting clonal expansion that influence infection risk, treatment tolerance, and survival.

Our results suggest that monitoring dynamic changes in CH during therapy, rather than baseline CH alone, could help identify patients at higher risk of adverse outcomes. Serial sampling during treatment may allow detection of expanding clones, particularly in genes such as *TP53* and *YLPM1* that show evidence of positive selection. Tracking this expansion during therapy may provide an early signal of emerging risk. Patients with CH mutations may therefore benefit from careful monitoring during cytotoxic therapies, particularly chemotherapy, as measurable clonal expansion can occur within the approximately 18-month timeframe captured by our analysis. These findings suggest a potential role for longitudinal CH monitoring as a complementary biomarker for guiding patient stratification during treatment. As tumor-informed minimal/measurable residual disease assays are now entering clinical practice, the presence of increasing CH could also be explored in prospective studies as a trigger to initiate or increase the frequency of plasma minimal/measurable residual disease testing to detect subclinical tumor recurrence.

Beyond cell-intrinsic genetic advantages, the presence and expansion of CH clones may also reflect interactions with the broader hematopoietic and inflammatory microenvironment. Emerging evidence indicates that CH-associated mutations can alter inflammatory signaling and reshape the bone marrow niche, generating feedback loops that further promote clonal expansion. Thus, therapy-related stressors such as chemotherapy or radiation may interact not only with the genetic properties of CH clones, but also with the surrounding inflammatory and hematopoietic microenvironment to shape clonal dynamics (8, 50, 52). Expanding CH clones seen in peripheral blood may also reflect an increasing presence of CH cells in the micrometastatic tumor microenvironment. As tumor-associated CH has been linked to worse outcomes in lung cancer (8), it raises the hypothesis that increasing peripheral CH may reflect enrichment of CH cells in the tumor microenvironment surrounding dormant cancer cells, which then may contribute to future outgrowth and activation and metastatic progression. Taken together, these observations suggest that CH may function both as a biomarker of hematopoietic stress and as a biologically active process influencing systemic inflammation and tissue environments. Longitudinal monitoring of CH during therapy could therefore provide clinically relevant insight into treatment-associated selective pressures and patient-specific risk. Work is ongoing to define the optimal timing, frequency, and actionable thresholds for such monitoring.

In conclusion, these findings demonstrate that both treatment type and dynamic changes in CH during therapy have important clinical implications. The evolutionary responses of blood clones to cancer treatment have direct translational relevance for predicting patient outcomes. While CH presence alone does not universally confer adverse risk, longitudinal changes in CH allelic burden during treatment may signal increased susceptibility to therapy-related complications across solid tumors. Further validation in larger, clinically homogeneous cohorts is required to substantiate these observations.

## Methods

### Sex as a biological variable.

This study exclusively included breast tumor samples from female patients (*n* = 171), consistent with the predominance of breast cancer in women. As such, sex was not evaluated as a biological variable, and the generalizability of these findings to male breast cancer remains unknown.

### Patient population, inclusion criteria, and clinical parameters.

Clinical data and samples used for this study were collected between January 1, 1994, and July 31, 2021. Patients were eligible for inclusion if they had a primary breast cancer diagnosis and serial peripheral blood samples available in the Moffitt Cancer Center’s institutional biorepository that could be used for DNA extraction and CH detection, including 1 sample collected before the start of any cancer treatment and 1 sample collected after the first therapy given for breast cancer. Specifically, the pretreatment sample needed to be collected within 1 year prior to the start of treatment or a maximum of 5 days after the start of treatment for patients given chemotherapy or hormone therapy. Patients treated with radiation needed to have a sample collected within 1 year before the radiation start date. For patients treated with chemotherapy, the first sequential sample needed to be collected after the chemotherapy stop date, within 1 year of the start date and before any radiation treatment. For patients treated with radiation, the first sequential sample needed to be taken at least 100 days after radiation, within 1 year of the start of radiation and before any chemotherapy treatment. For patients treated with hormone therapy, the first sequential sample had to be collected within 1 year, but at least 85 days (the average duration of chemotherapy), after the start date of hormone therapy and before any exposure to chemotherapy or radiation. For patients meeting these inclusion criteria, all other serial sampling time points available in the institutional biorepository, including during treatment and after the post-treatment sampling times, were included.

Patients’ cumulative exposure to chemotherapy drugs and radiation during the observation period was calculated following the approach by Bolton et al. (15), derived from the Late Effects Study Group 1 (53). For each patient, the cumulative dose per kilogram for each drug received during the observation period was calculated, converted into tertile-based scores, and summed within drug classes. Radiation dose tertiles were calculated using the cumulative radiation dose during the observation period in 2 Gy per fraction (EQD_2_), using an α/β of 4 Gy (54). These class-level scores were then divided into tertiles to categorize overall exposure across the cohort. Of note, cumulative radiation exposure was largely similar across the population (EQD_2_ median, IQR).

### DNA extraction and hybrid-capture, error-corrected sequencing.

DNA was extracted using the Autopure LS automated DNA extractor (QIAGEN) and quantified using Qubit (Invitrogen, Thermo Fisher Scientific). Molecular data generation was conducted in collaboration with the Moffitt Molecular Genomics Core. DNA libraries were constructed using a custom-designed SureSelect^XTHS2^ kit (Agilent Technologies), which optimally captured whole exons of 81 hematopoietic disorder–related genes, including single nucleotide mutations and small indels. Libraries were sequenced on a NextSeq 2000 sequencer (Illumina), per the manufacturer’s recommendations, with a goal of achieving an average depth of coverage of greater than 5,000×.

### Bioinformatics analysis and somatic variant calling.

Sequencing reads were aligned to a corrected human genome (GRCh38) reference (55) using the BWA-MEM algorithm (56). Consensus variant calling from reads with the same unique molecular identifiers was done using fgbio version 2.2.1 (57). Somatic variant calling was performed using Genome Analysis Toolkit best practices and Mutect2 (58). Additional statistical support for the detection of variants in individual samples was obtained using a β-binomial model for allele-specific sequencing noise (FDR <1 × 10^–4^) (59). Variants were filtered and annotated using BCFtools (60). Insertions and deletions were called by 2 indel callers, Mutect2 and Lofreq (61), and were only retained if the VAF was 0.02 or higher. Variants that occurred in greater than one-tenth of the samples were considered sequencing artifacts and were removed. Quality control filters included a strand OR of less than 3, a quality score of greater than 6.3, an observation of each variant at least once on the forward and reverse reads, and a masking of the repetitive regions of the genome as defined by the DUST algorithm (62) plus a region with low complexity in *KMT2D* (c. 1659–2544) linked to unreliable calling. All analyses in this study were performed using the full set of detected CH mutations (VAF >0.01%); the term CHIP (VAF ≥2%) is used only in analyses specifically restricted to clinically defined CHIP-level mutations.

Germline variants were removed using publicly available reference populations (i.e., variants observed in noncancer populations at a prevalence of >0.005 and with a VAF of >0.25) (63, 64). The high sequencing depth in this study yielded very small CIs of less than 0.5% for measuring VAF; therefore, germline variants that are expected to be present at 50% allele frequency were robustly identified (65); however, to ensure removal of private germline variants, we excluded variants with a VAF of greater than 40% detected in each patient’s serial samples. To filter for likely functional somatic variants, only mutations or indels located in exonic regions were considered CH mutations. Germline SNP loci heatmaps were used to confirm correct sample pairing within patients. Chart review confirmed nontumor cell origin of *TP53* CH mutations and distinction from *TP53* mutations detected by prior sequencing of tumor specimens.

### External validation cohorts.

The mutational and clinical data for the DFCI and MSKCC cohorts were obtained through data transfer agreements between the institutions or from their respective publications (5, 22) and were reviewed to match the patient selection and variant calling criteria in the discovery cohort. In particular, downstream analyses were limited to cases with CH VAFs measured before and after cancer treatment and those with exonic variants curated by Vlasschaert et al. (66).

### Clonal selection of CH driver genes.

Genes with driver CH mutations were expected to show a higher number of loss-of-function nonsense or hotspot missense mutations compared with the expected distribution under neutral selection. χ^2^ Statistics were used to compare the observed to expected mutations counts, and lower and upper 95% binomial-approximated CIs were calculated to assess uncertainty for excess of nonsense, missense, and synonymous mutations.

### Calculating standardized CH VAF change per time.

VAFs were quantified across serial samples, and *Z* statistics were used to measure temporal mutation- and gene-specific VAF changes. Changes in CH allele frequencies for mutation *j* in patient *i* were normalized and defined by 
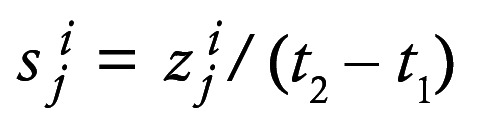
, where *t_1_* and *t_2_* were collection time points, and 
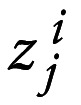
 was a 2-proportion *Z* test. Across all analyses, the mean and standard error in the distributions of VAF changes were used to calculate a standardized VAF change per unit time and its CIs for specific treatments and genes (per treatment modality).

In more detail, a 2-proportion *Z* test was used to compare CH VAFs between time points, accounting for differences in sequencing depths. The *Z* statistic was calculated on the basis of the VAFs (*f_1_* and *f_2_*) and total sequencing depths (*D_1_* and *D_2_*) measured in serial samples, following 

(Equation 1) 
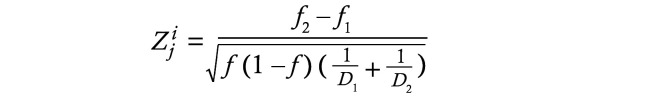


with 

(Equation 2) 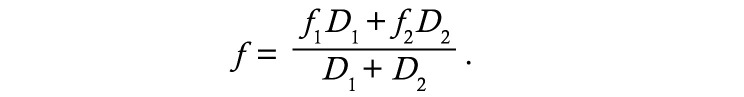


The pooled allele frequency *f* is bounded within [0,1] and represents a weighted average of 2 proportions. In this context, the null hypothesis is that standardized CH VAFs do not change during treatment, and as such, *H*_0_:*f*_1_ = *f*_2_. Since sequencing depths differ in each VAF measurement, this *Z* statistic reflects the changes in VAFs relative to sampling noise beyond just the raw measurement. For example, a small VAF change at very high depth leads to a large *Z*, and a large VAF change at low depth results in a small *Z*, driven by statistical certainty and not the VAF change magnitude alone.

The 95% CIs for standardized VAF change estimates were calculated using CIs for 
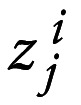
 at α = 1.96. The standard deviation of the *Z* statistics is given by 

(Equation 3) 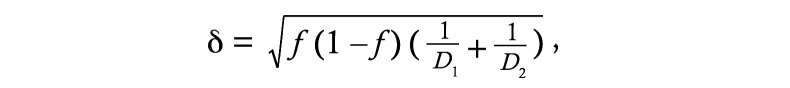


which for the fixed allele frequencies *f_1_* and *f_2_*, converges to zero as sequencing depths *D_1_* and *D_2_* increase and the term

(Equation 4) 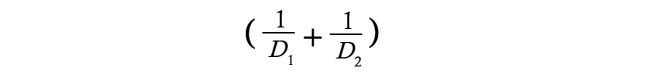


converges to zero. Finally, since 

(Equation 5) 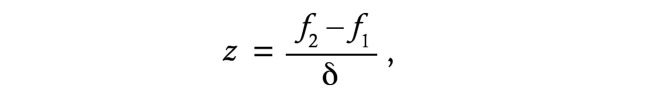


any non-zero allele frequency difference yields increasingly large *Z* values with increasing depths. Thus, this metric reflects statistical confidence in VAF change and is depth-aware by definition.

Therefore, the resulting *Z* statistic, normalized by elapsed time, was used to define a metric to calculate statistical confidence for changes in CH VAFs while considering sampling variance. As detailed above, this *Z* statistic scales with sequencing depth, and therefore 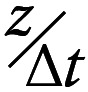 represents a significance-weighted, time-normalized statistical measure of change in VAF that incorporates uncertainty arising from finite sampling. It should be noted that this metric cannot be interpreted as a direct estimate of biological growth rate or selective advantage.

### Therapeutic bottleneck and evolutionary fitness likelihood calculation.

A binomial sampling model was applied to estimate effective hematopoietic cell population size and CH fitness likelihood during treatment. To estimate the effective allelic population size, it was assumed that CH mutations were clonally independent and that VAFs were representative of single CH clones. If *n_i_* mutated alleles harbored a specific variant at time point 1, the probability of observing *m_i_* mutated alleles with the same variant at time point 2 would be described by binomial sampling 

(Equation 6) 
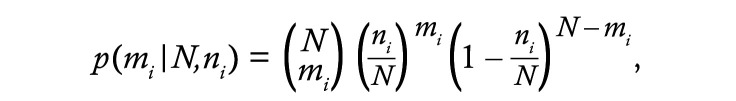


 with bottleneck size *N*. For the *s* number of CH variants shared between any 2 time points, the maximum likelihood estimate for *N*, describing the lower bound on effective bottleneck population size was equal to 

(Equation 7) 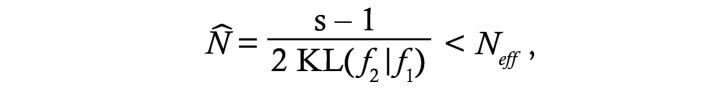


 with KL representing the Kullback-Leibler divergence, and *f_1_* and *f_2_* as measured VAFs at time points 1 and 2, respectively. The maximum likelihood–estimated variance of *N_eff_* was then equal to 
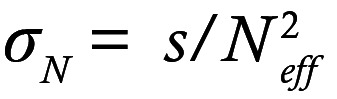
.

Using patient-specific effective allelic population size (*N_eff_*) estimates, the probability of observing a post-treatment VAF (*f*_2_) given an initial pretreatment VAF (*f*_1_) under neutral drift was modeled using a binomial sampling process: 

(Equation 8) 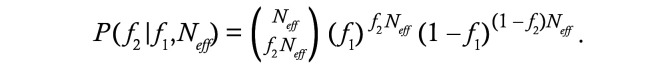


This represents the likelihood that the observed allele frequency change can be explained by stochastic genetic drift alone following a treatment-induced population bottleneck. The evolutionary fitness likelihood for each mutation was then defined as the negative base 10 logarithm of this probability: *L* = –log_10_(*P*(*f*_2_|*f*_1_,*N_eff_*)). Operational classification of selection was defined as follows: neutral: *L* ≤ 5 (VAF change consistent with drift); positive selection: *L* > 5 and *f_2_* > *f_1_*; negative selection: *L* > 5 and *f_2_* < *f_1_*. Thus, selection reflects statistically unlikely deviation from neutral drift expectations rather than raw VAF change alone.

### Depth sensitivity analysis.

The observed VAF was derived from finite read depth and thus subject to sampling noise, and the high sequencing depth in our dataset (mean >5,000× per mutation) ensured that the binomial sampling variance was small relative to the observed differences in allele frequency. Therefore, treating the observed VAF as an exact probability provided a close approximation to the true underlying frequency. Any residual sampling noise would tend to bias the effective population size (*N_eff_*) estimates downward, making our estimates conservative. Furthermore, since read depths were comparable across mutations and patients, the relative differences in *N_eff_* between clones and individuals remained valid, supporting the conclusions drawn from our analysis. To quantify these expectations, we calculated the CI of *N_eff_* on the basis of the propagation of uncertainty in measuring VAFs as a function of sequencing depth.

### Sampling variance for CH VAFs.

Observed VAFs are affected by sequencing sampling noise. For a sequencing depth of *D*, the sampling variance of allele frequencies *p_i_* and *q_i_* are approximated by a binomial model: 

(Equation 9) 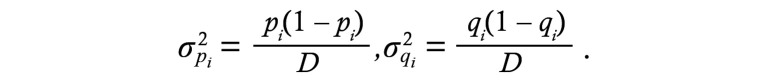


Since the frequency of the WT clone is equal to 1 minus the sum of CH VAFs, the variance in WT frequency is equal to the sum of variances in CH VAFs. Thus, the uncertainty in *N_eff_* depends on sequencing depth through the uncertainty in measured allele frequencies.

### Propagation of Uncertainty for N_eff_.

Effective population size *N_eff_* can be estimated with 

(Equation 10) 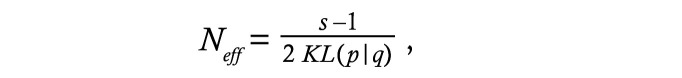


where 

(Equation 11) 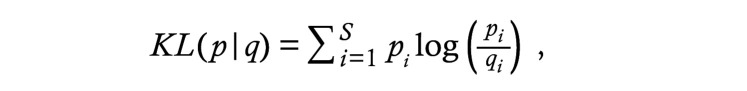


where *s* is the number of CH and WT clones, and *q* and *p* are their pre- and post-treatment allele frequencies, respectively. To estimate the uncertainty of *N_eff_*, a first-order error propagation was applied where the variance of was approximately equal to 

(Equation 12) 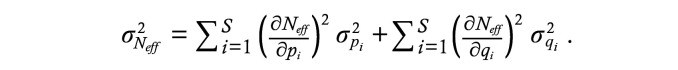


The derivatives followed from the dependence of *N_eff_* on the KL divergence: 

(Equation 13) 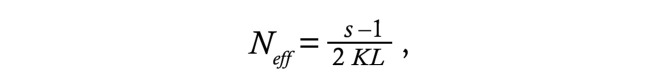


so that 

(Equation 14) 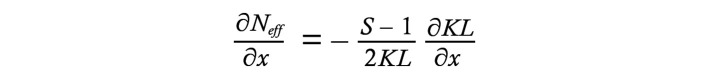


for *x* ∈ {*p_i_*,*q_i_*}. The derivatives of the KL divergence are 

(Equation 15) 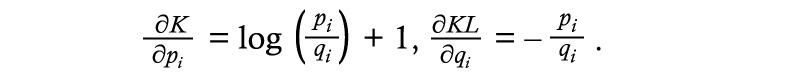


Substituting these derivatives into the propagation formula yields an analytical approximation of the variance of *N_eff_* as a function of sequencing depth. CIs for *N_eff_* could then be approximated using 
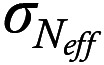
, where 95% CIs equal to 
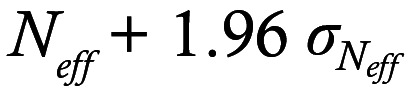
.

To evaluate the effect of sequencing depth on the precision of the *N_eff_* estimator, the above propagation was evaluated across a range of sequencing depths 200× to 6,000×. For each patient in our cohort, we downsampled the sequencing depths and, for each depth, calculated the sampling variance for measured CH VAFs according to the model above, yielding depth-dependent CIs for *N_eff_*. As expected, the coefficient of variance calculated across the cohort for all patients showed a reduction of error in estimating *N_eff_* at higher depths (Supplemental Figure 10).

### Clonal independence sensitivity analysis.

In estimating the *N_eff_*, it was assumed that each CH mutation marked an independent clone. While this assumption is supported by the low biological plausibility of sequential CH-driving mutations arising within the same cell as discussed in Marzban et al. (67), nested or co-occurring clones may occur as predicted by Watson et al. (10). To assess the effect of nested clones on *N_eff_* estimation, all analyses were repeated, assuming that all detected CH variants were nested in the largest clone, representing the most restrictive scenario. This analysis showed that the differences in *N_eff_* across treatment groups, as well as CH mutational dynamics, remained consistent with our original analyses (Supplemental Figure 10).

### Statistical and clinical association analyses.

Statistical significance between groups was assessed using exact tests, χ^2^ tests, log-rank tests, or nonparametric rank-sum tests, as indicated. The Benjamini-Hochberg FDR method was applied to adjust for multiple hypotheses testing. Clinical associations were evaluated using multivariable Cox proportional hazards regression models assessing OS and PFS. Models included age, cancer stage, and ER, PR, and HER2 status as covariates. Because CH selection status was defined using post-baseline measurements (with baseline defined as the treatment start date), potential concerns about immortal time bias were addressed by landmark analyses at 12 and 18 months, as well as a time-dependent Cox model incorporating patient-specific sample collection time points. Given the limited number of covariates relative to the number of events, the models fell within commonly accepted ranges for events per variable (≥10 events per variable), reducing the likelihood of substantial overfitting. Statistical analysis and data visualizations were performed using the following packages in R: surminer, survival (survival analyses), and ggplot2 (plotting). Maftools (68) was used for generating oncoplot and lollipop figures, and EvoFreq (69) was used to visualize the evolutionary models.

### Study approval.

Patients for this study were consented to the Moffitt Cancer Center’s Total Cancer Care Protocol, an IRB-approved institutional biorepository (MCC#14690; Advarra IRB Pro00014441) (70). Use of biobanked patient samples for genetic data generation for this study was approved under a release protocol (MCC#21545, Advarra IRB Pro00058968).

### Data availability.

Raw sequencing data on peripheral blood from the discovery cohort (*n* = 171 patients) were deposited in the dbGaP repository (phs004283.v1.p1). All data used in the figures and throughout the manuscript are provided in the Supporting Data Values file and Supplemental Tables.

### Code availability.

All code to reproduce and calculate the evolutionary fitness and selection pressure strength is available on GitHub at https://github.com/marabzadeh/EchoCH

## Author contributions

NG, HK, and SG conceived and supervised the study. MA performed evolutionary modeling. MA and YHT conducted sequencing analyses, variant calling, statistical association analyses, and visualization of the results with help from VM and MT. CCL and JKK assisted with identifying the patient cohort. CCL extracted and analyzed clinical data with help from DW, EH, and IW. S Marzban visualized therapeutic bottleneck results with supervision from JW. S Morganti, HAP, and JEG generated and analyzed the DFCI cohort data. All authors drafted the manuscript, and all authors read and approved the final manuscript.

## Conflict of interest

SG has been a consultant for KayoThera, Lunit, Ipsen, Roche, Merck, Foghorn Therapeutics, and EQRX and has received research funding from Gandeeva and M2GEN. S Morganti reports serving in consulting or advisory roles at Daiichi-Sankyo and receiving institutional research funding from Precede Biosciences and Merck. HK is a full-time employee of Regeneron Pharmaceuticals.

## Funding support

This manuscript is the result of funding in whole or in part by the NIH. It is subject to the NIH Public Access Policy. Through acceptance of this federal funding, the NIH has been given a right to make this manuscript publicly available in PubMed Central upon the Official Date of Publication, as defined by the NIH.

- National Cancer Institute (NCI), NIH (R01-CA233662, to HK, SG, and NG).
- Collaborative Data Services Core Facility, Tissue Core Facility, Molecular Genomics Core Facility, and Total Cancer Care Protocol at the H. Lee Moffitt Cancer Center and Research Institute (P30-CA076292, to NG).
- Florida Breast Cancer Research Foundation (to NG, JW).
- Rutgers Cancer Institute Biomedical Informatics Shared Resource (P30-CA072720-5917, to HK and SG).
- New Jersey Commission on Cancer Research (COCR24PDF008, to MA).
- American-Italian Cancer Foundation, Fondazione Gianni Bonadonna, and the Associazione Italiana per la Ricerca contro il Cancro, Saverin Family Fund (to S Morganti).
- NYU Perlmutter Cancer Center Support Grant (P30-CA016087, to SG).

## Supplementary Material

Supplemental data

Supplemental table 1

Supplemental table 2

Supplemental table 3

Supplemental table 4

Supplemental table 5

Supplemental table 6

Supporting data values

## Figures and Tables

**Table 1:**
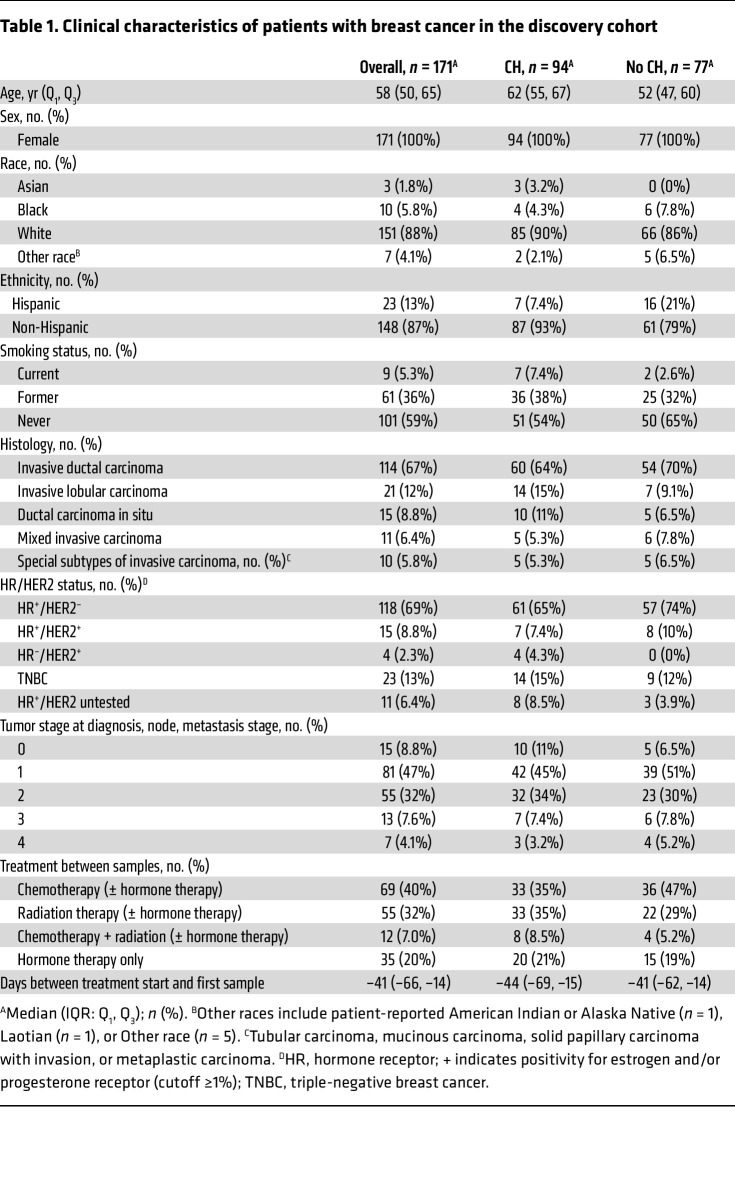
Clinical characteristics of patients with breast cancer in the discovery cohort

## Version 1 06/09/2026

In-Press Preview

## Version 2 07/15/2026

Electronic publication
