# Supplementary material for: Evolution of clonal hematopoiesis during cancer treatment and its impact on outcomes
Source: J Clin Invest. 2026 Jun 9;136(14):e204429. doi: 10.1172/JCI204429 (PMC13367969; doi:10.1172/JCI204429)
Supplement: Supplemental data [file jci-136-204429-s234.pdf]

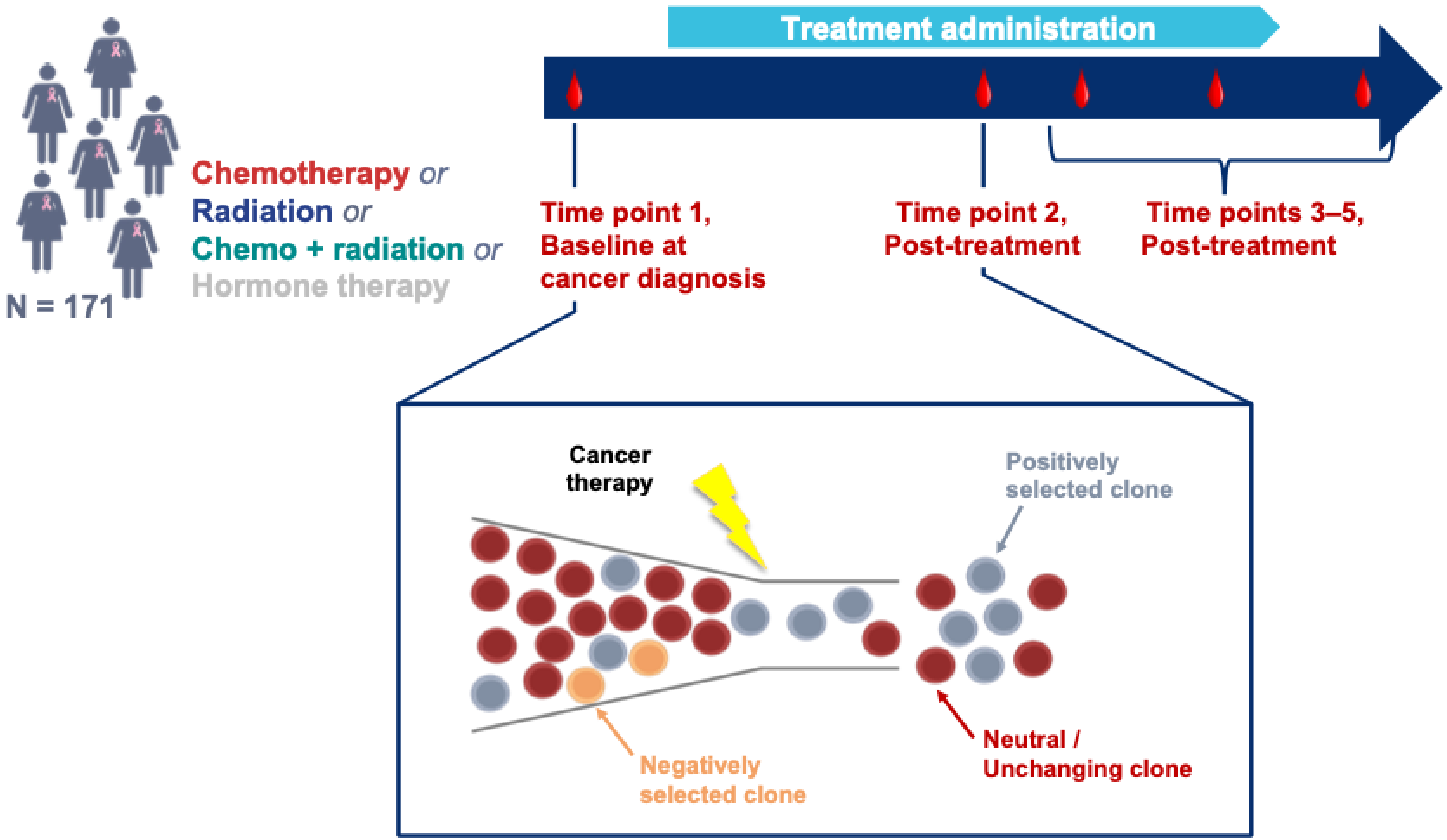

FIGURE S1. Schematics showing the study design, sampling timeline criteria, and evolutionary models considered for CH during breast cancer treatment.

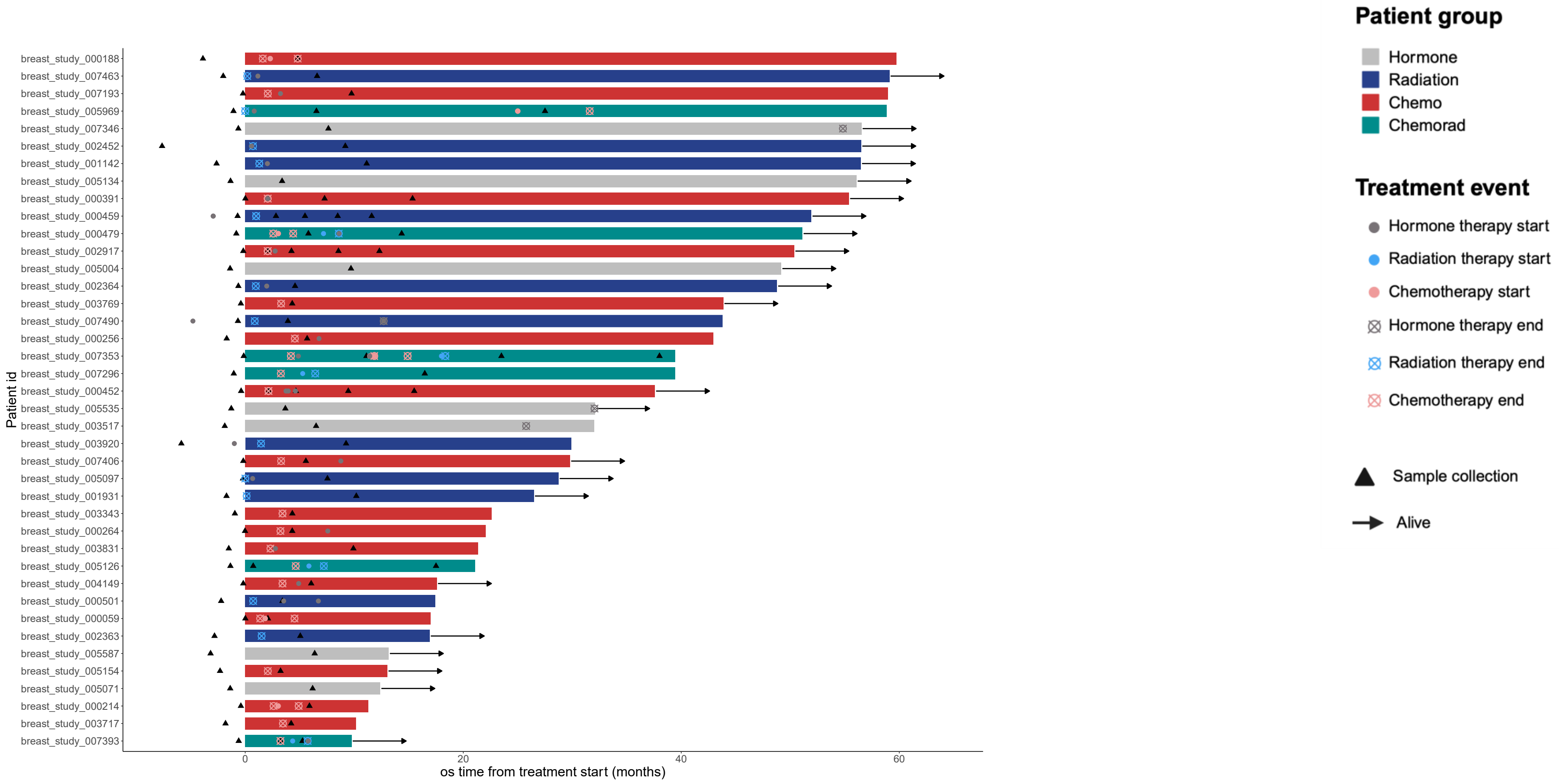

FIGURE S2A. Swimmer plots showing the sampling timeline relative to breast cancer diagnosis and treatment schedule per patient. Patients with overall survival (OS) ≤ 60 months are included.

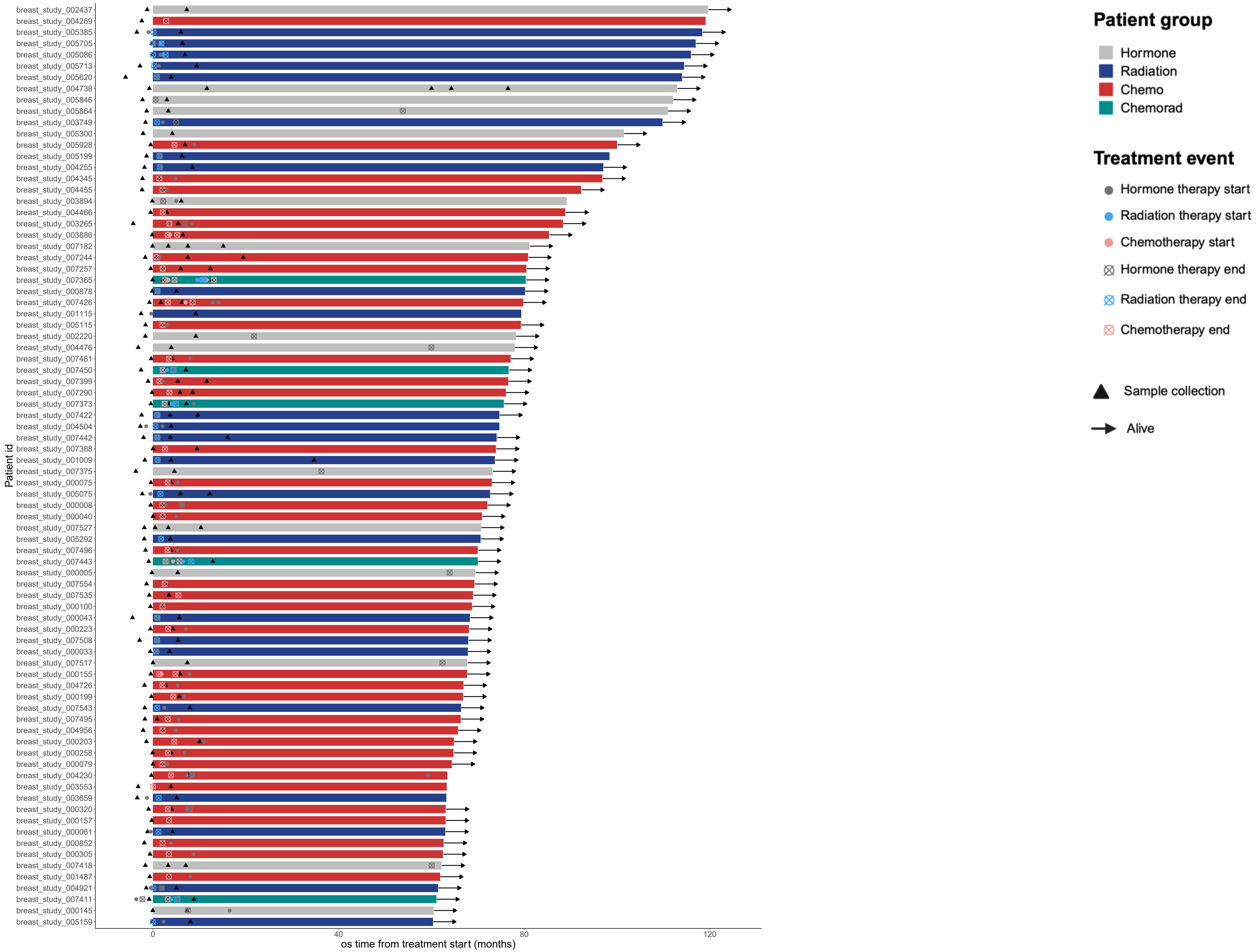

FIGURE S2B. Swimmer plots showing the sampling timeline relative to breast cancer diagnosis and treatment schedule per patient. Patients with overall survival (OS) 60-120 months are included.

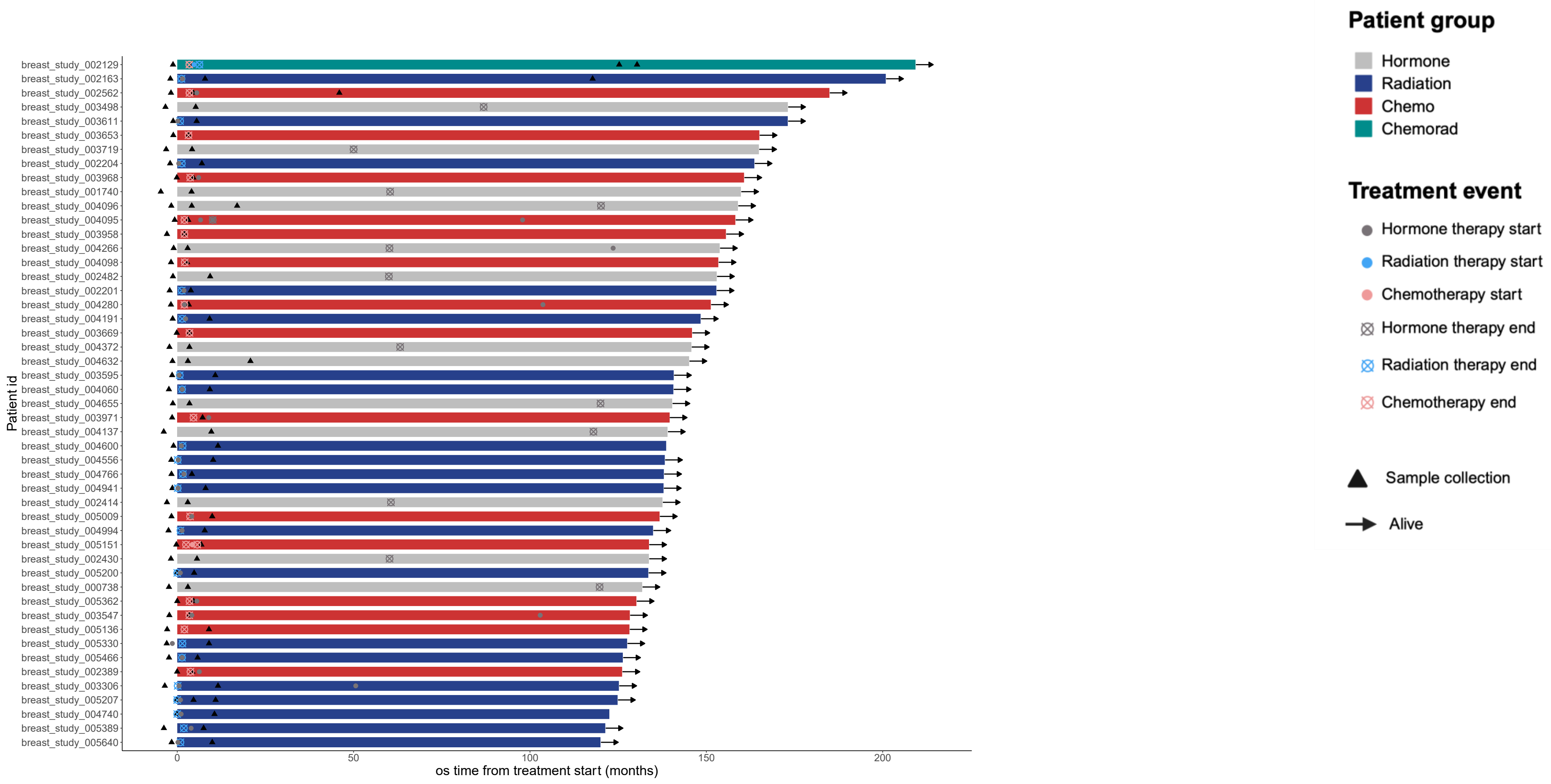

FIGURE S2C. Swimmer plots showing the sampling timeline relative to breast cancer diagnosis and treatment schedule per patient. Patients with overall survival (OS) > 120 months are included.

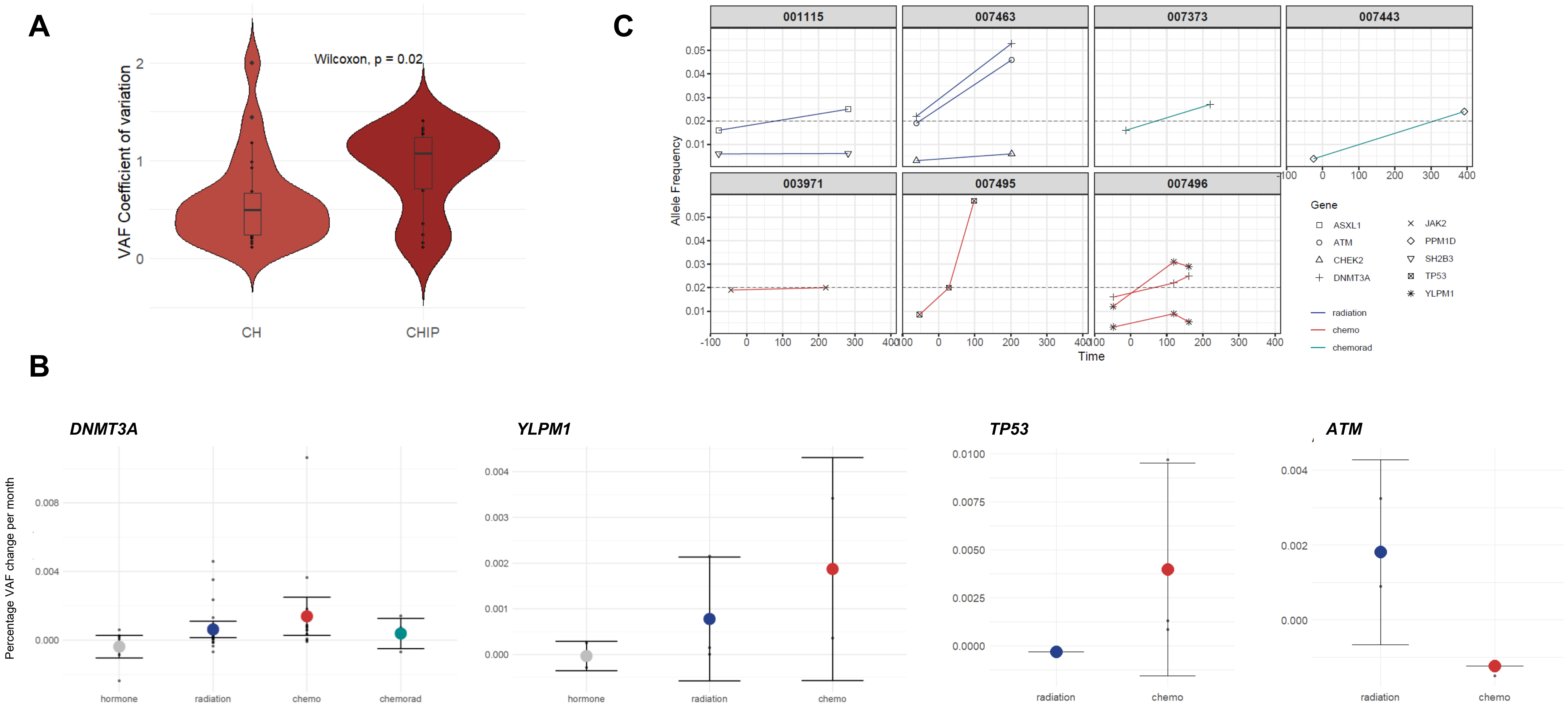

FIGURE S3. Clonal diversity and CH mutation-specific changes under treatment for breast cancer. A) Clonal diversity after excluding mutations with VAF ≥ 2% for both with and without CHIP-defined mutations groups. B) Standardized percent change in variant allele frequency (VAF) per month for *DNMT3A*, *YLPM1*, *TP53* and *ATM* mutations by treatment modality. C) Change in VAF for CH mutations that grow to CHIP-defining VAF during treatment.

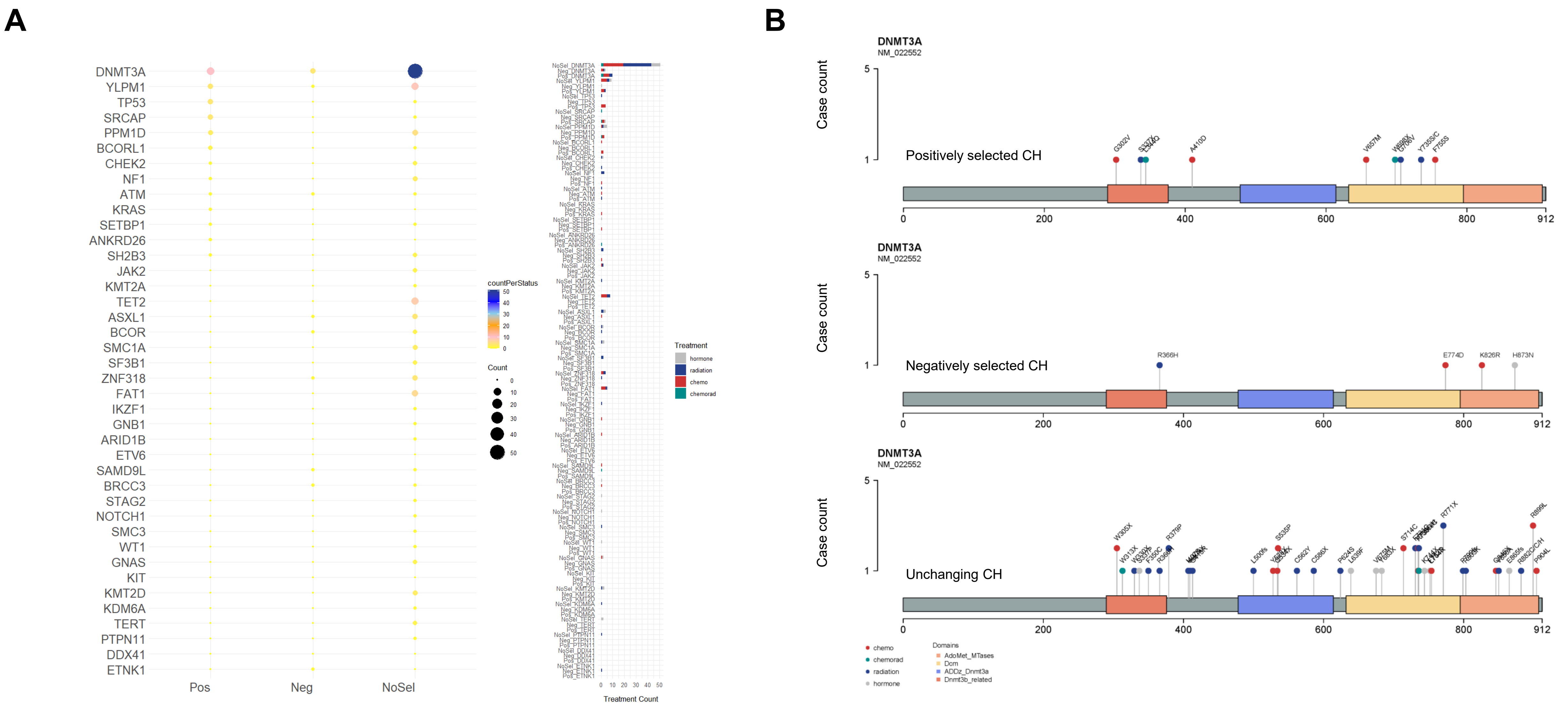

FIGURE S4. CH dynamics spectrum. A) The number of patients with positive (Pos), negative (Neg), or no selection (NoSel) of CH mutations across treatment modalities and genes. B) Mutational domain spectrum of DNMT3A in the 3 groups of negatively selected, positively selected, or unchanging CH.

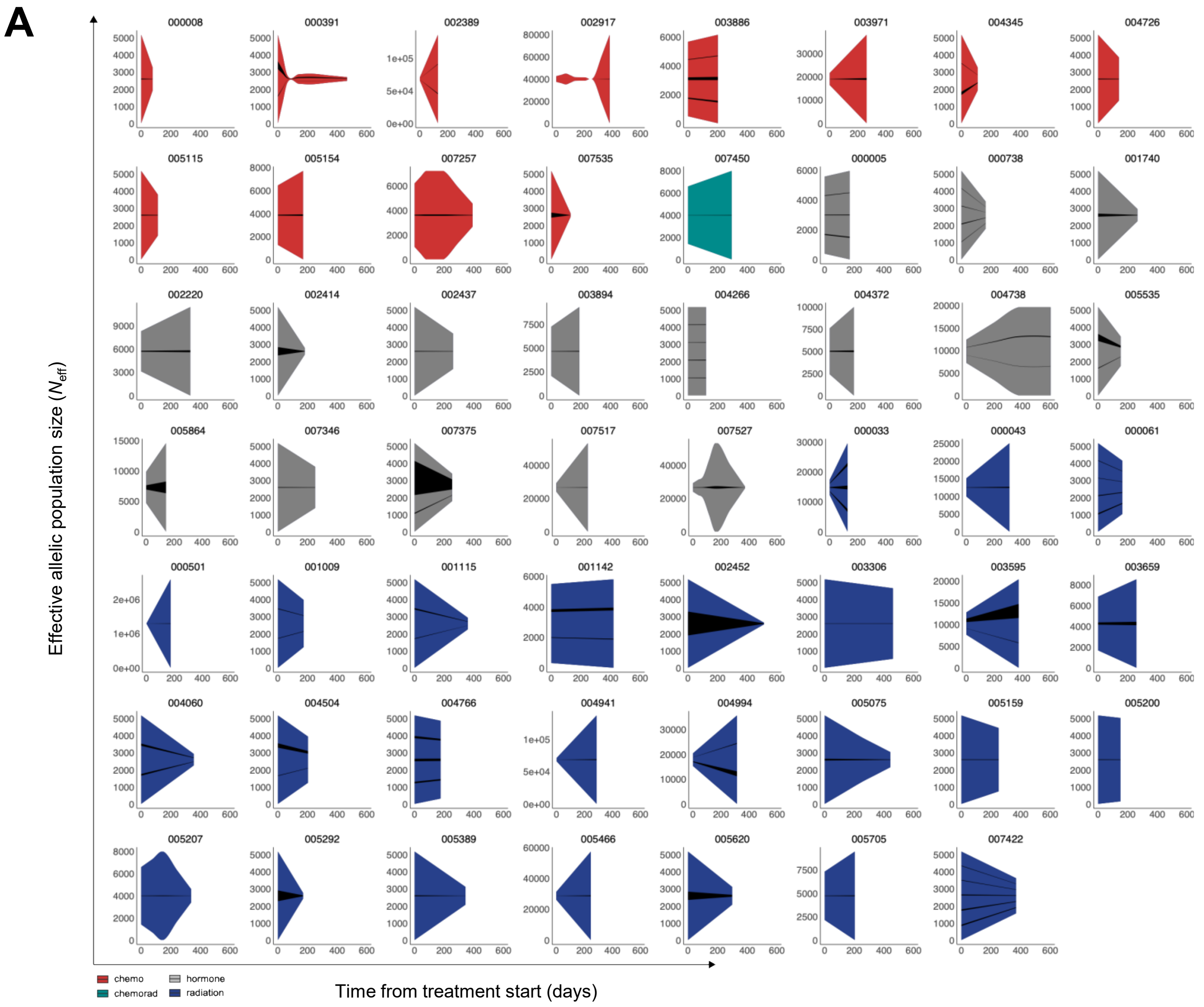

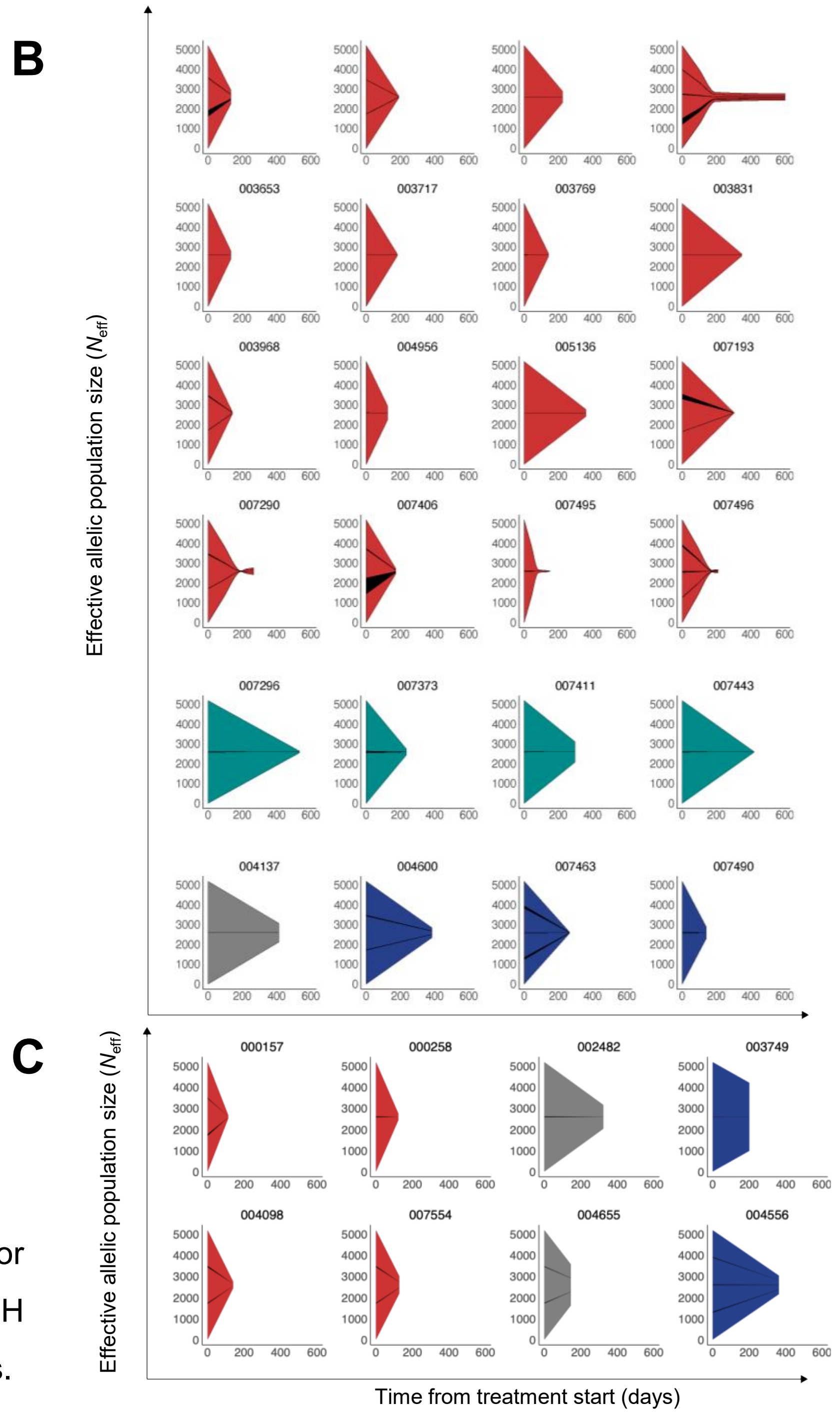

FIGURE S5. Schematics showing effective allelic population size (N_{eff}) during treatment for patients with A) unchanging CH, B) positively selected CH, or C) negatively selected CH normalized by mean N_{eff} in cases treated with hormonal therapy only, across treatment modalities.

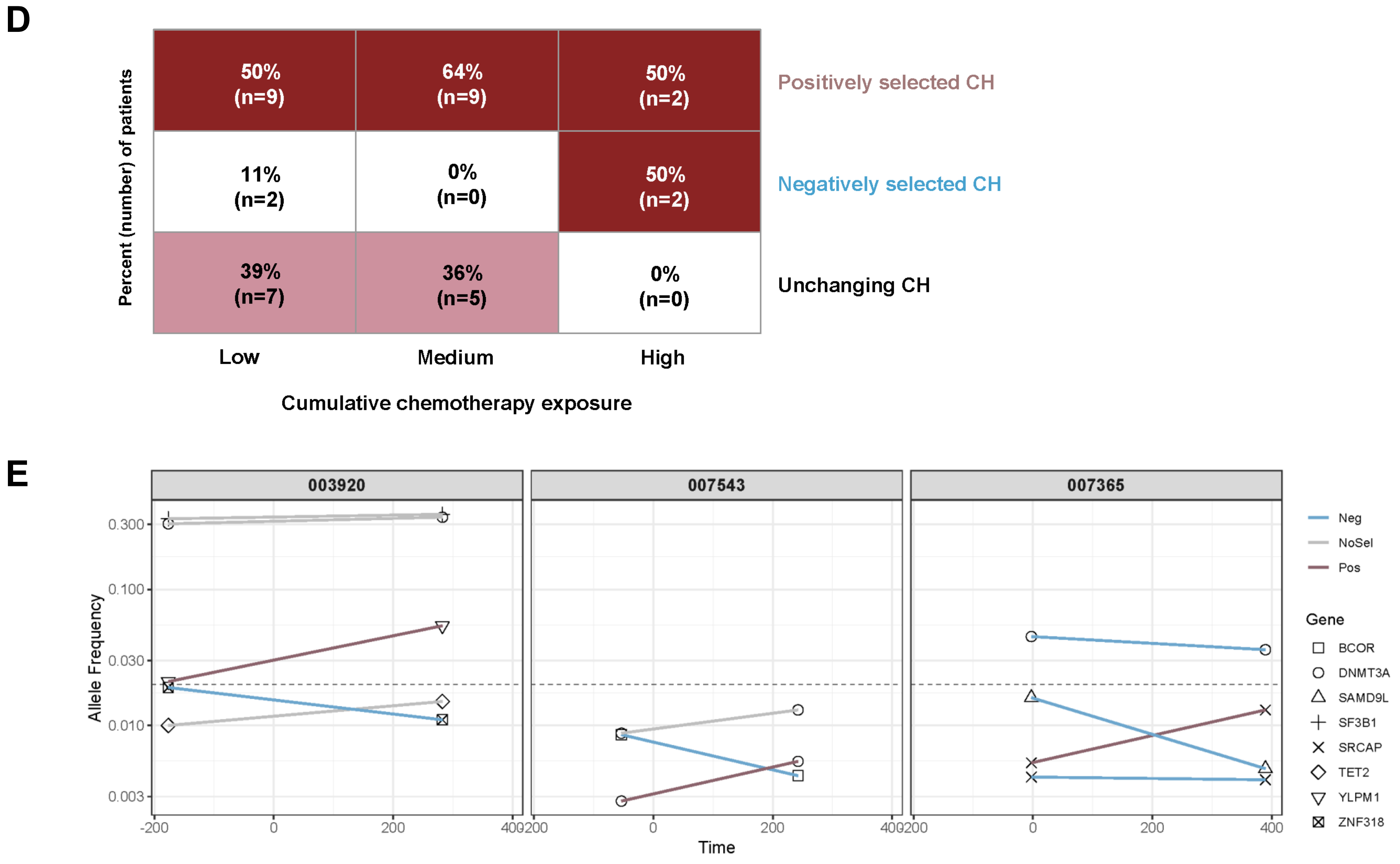

FIGURE S5. D) Percentage and number of patients across cumulative chemotherapy exposure levels stratified based on CH mutational dynamics. E) Change in VAF for CH mutations in patients with both positive and negative selection.

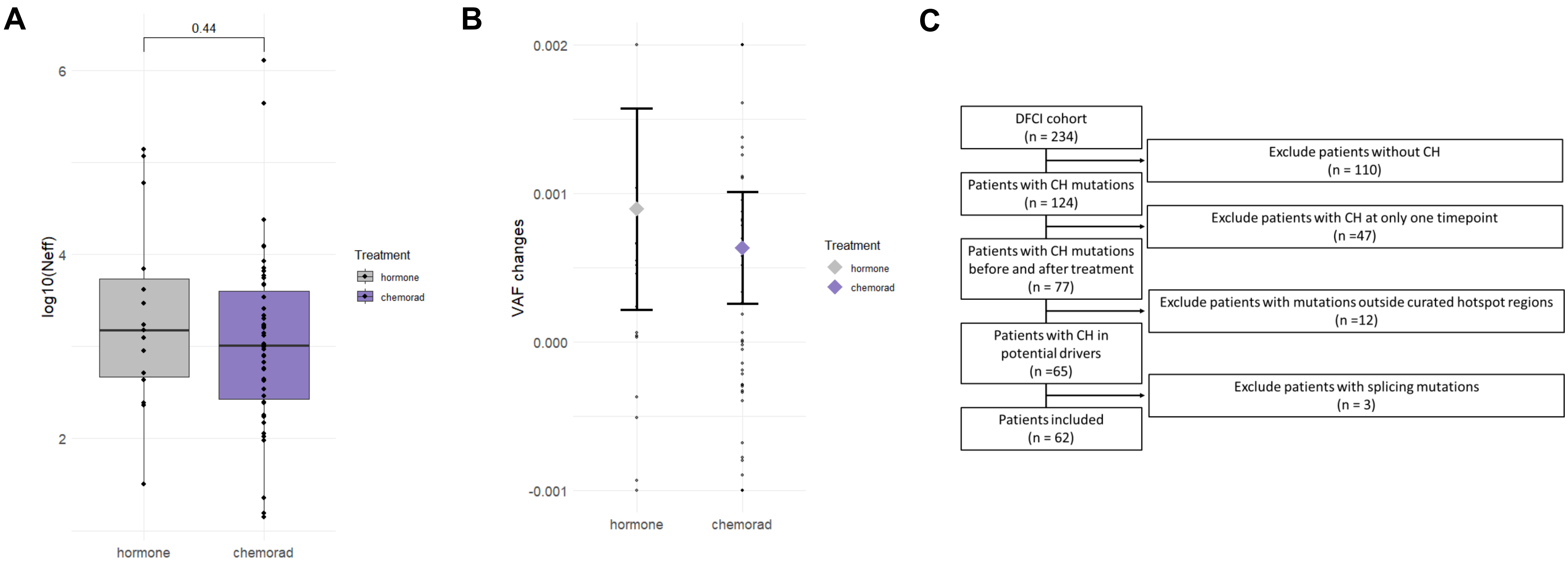

FIGURE S6. CH mutational dynamics in the Dana Farber Cancer Institute early-stage breast cancer cohort. A) Effective allelic population size (N_{eff}) across treatment modalities. B) Percent change in variant allele frequency (VAF) per month for CH mutations by treatment modality. C) REMARK diagram for the DFCI cohort.

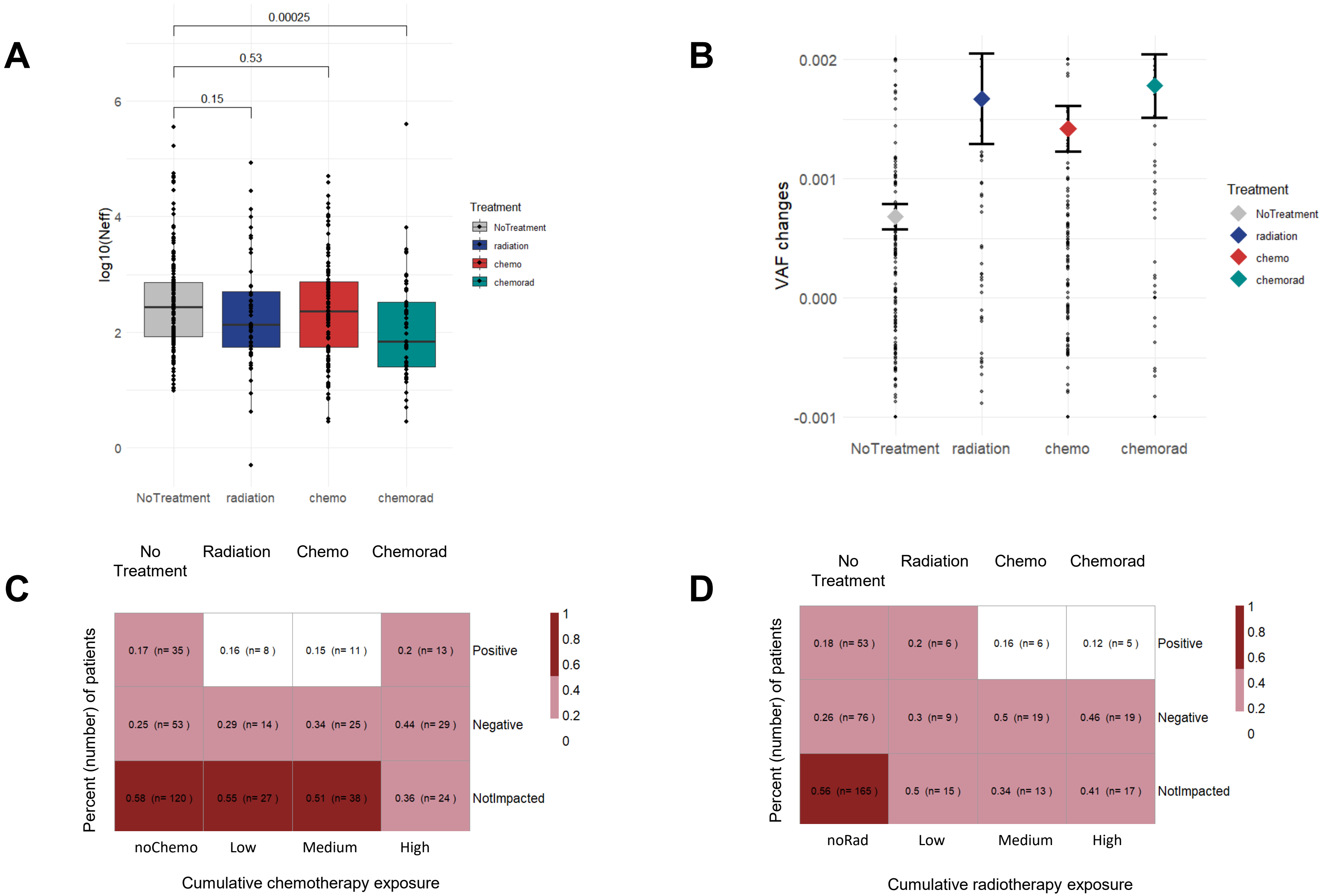

FIGURE S7. CH mutational dynamics in the Memorial Sloan Kettering Cancer Center pan-cancer cohort. A) Effective allelic population size (N_{eff}) across treatment modalities. A) Percent change in variant allele frequency (VAF) per month for CH mutations by treatment modality. C) Number and percentage of patients across cumulative chemotherapy exposure levels stratified by CH mutational dynamics. D) Number and percentage of patients across cumulative radiotherapy exposure levels stratified by CH mutational dynamics.

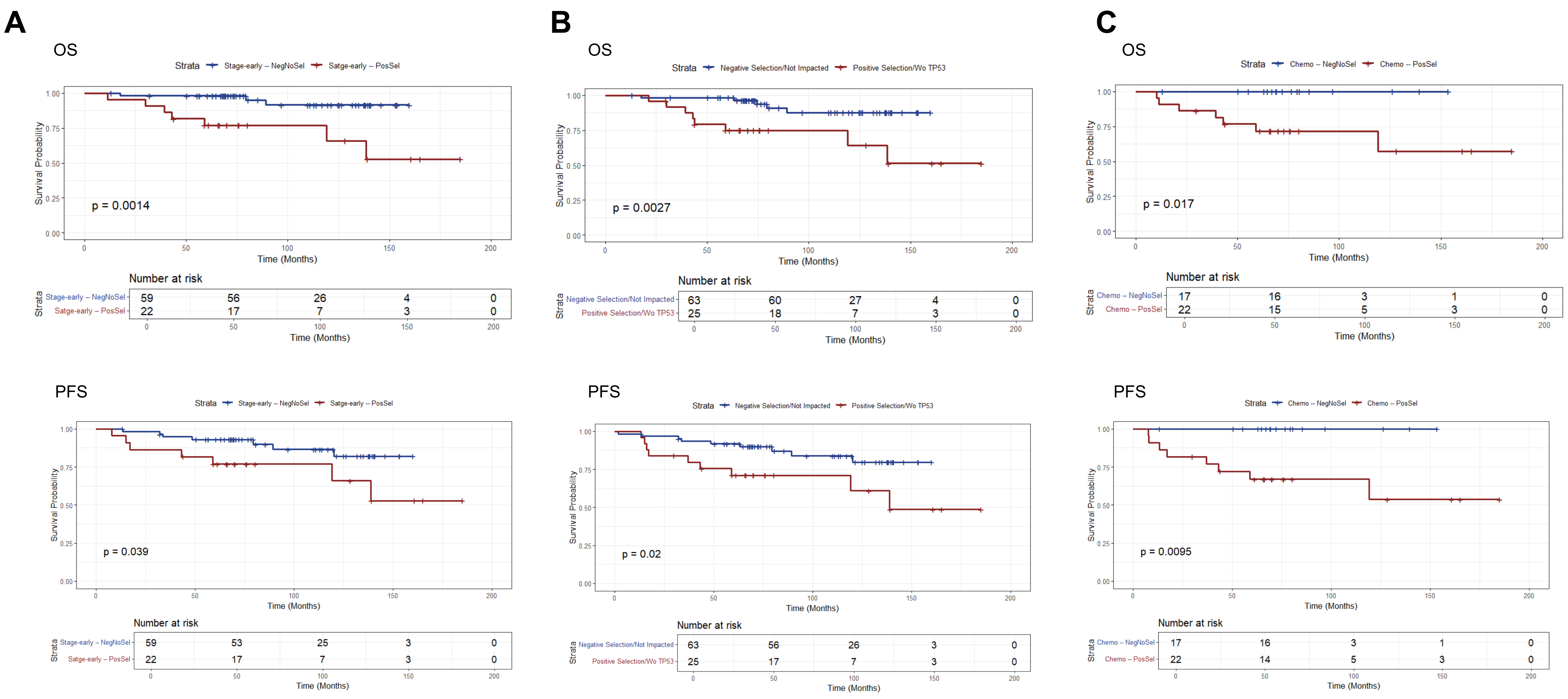

FIGURE S8. Difference in overall survival (OS) and progression free survival (PFS) between patients with positively selected CH and those with either negatively selected or unchanging CH. A) Including only patients with early-stage disease. B) Excluding patients with TP53-mutated CH. C) Including patients treated only with chemotherapy during examined period.

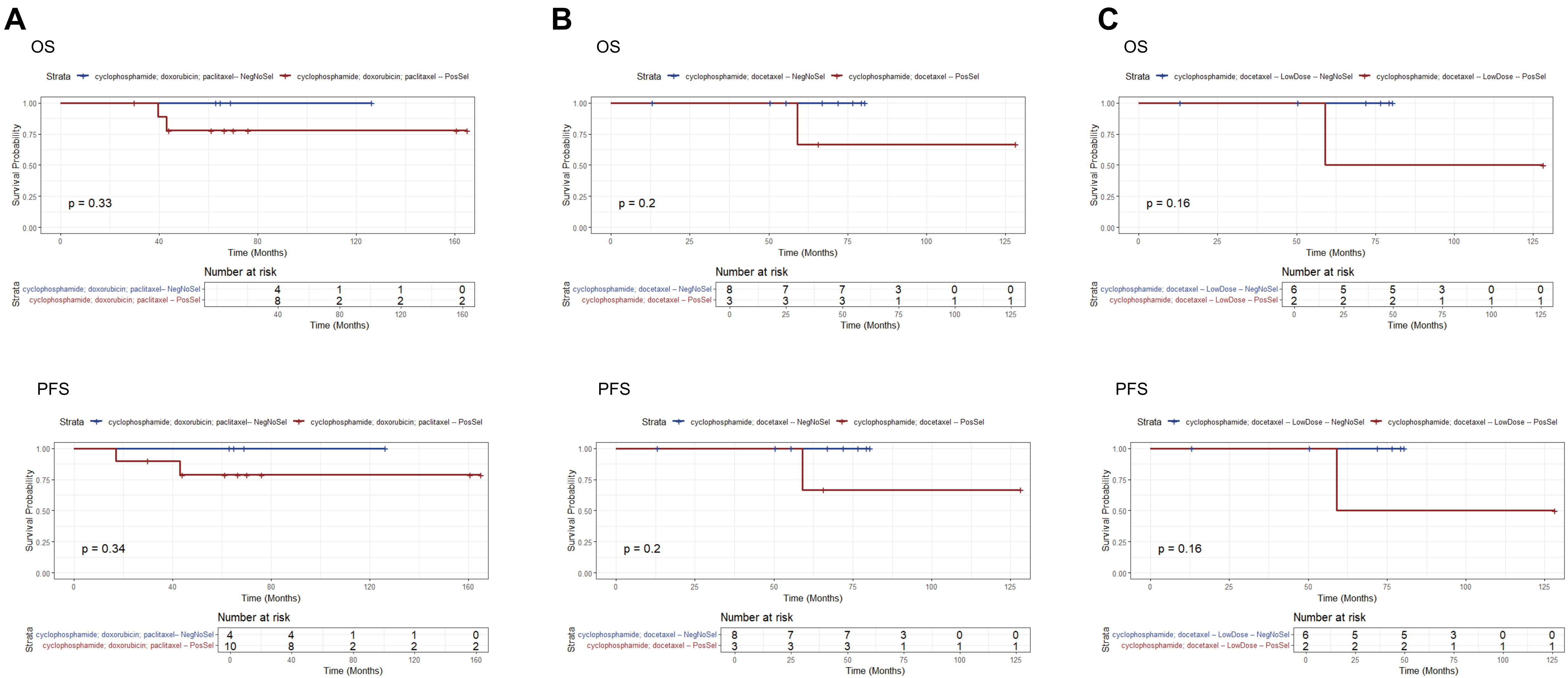

FIGURE S9. Difference in overall survival (OS) and progression free survival (PFS) between patients with positively selected CH and those with negatively selected or unchanging CH in subsets of the cohort under homogenous treatment. A) Including patients receiving cyclophosphamide, doxorubicin, and paclitaxel (n=14). B) Including patients receiving cyclophosphamide and docetaxel (n=11). C) Including patients receiving low levels of cyclophosphamide and docetaxel (n=8).

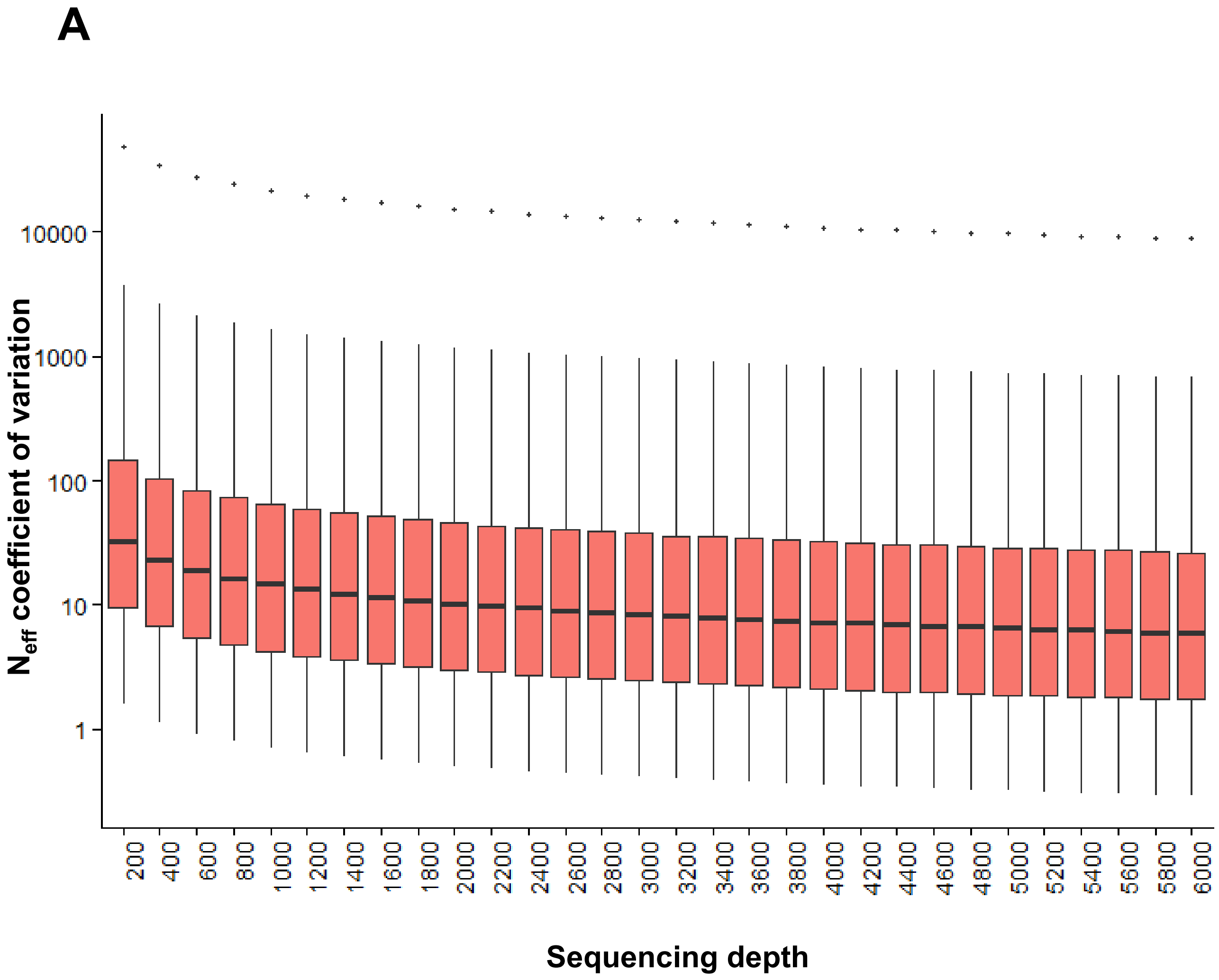

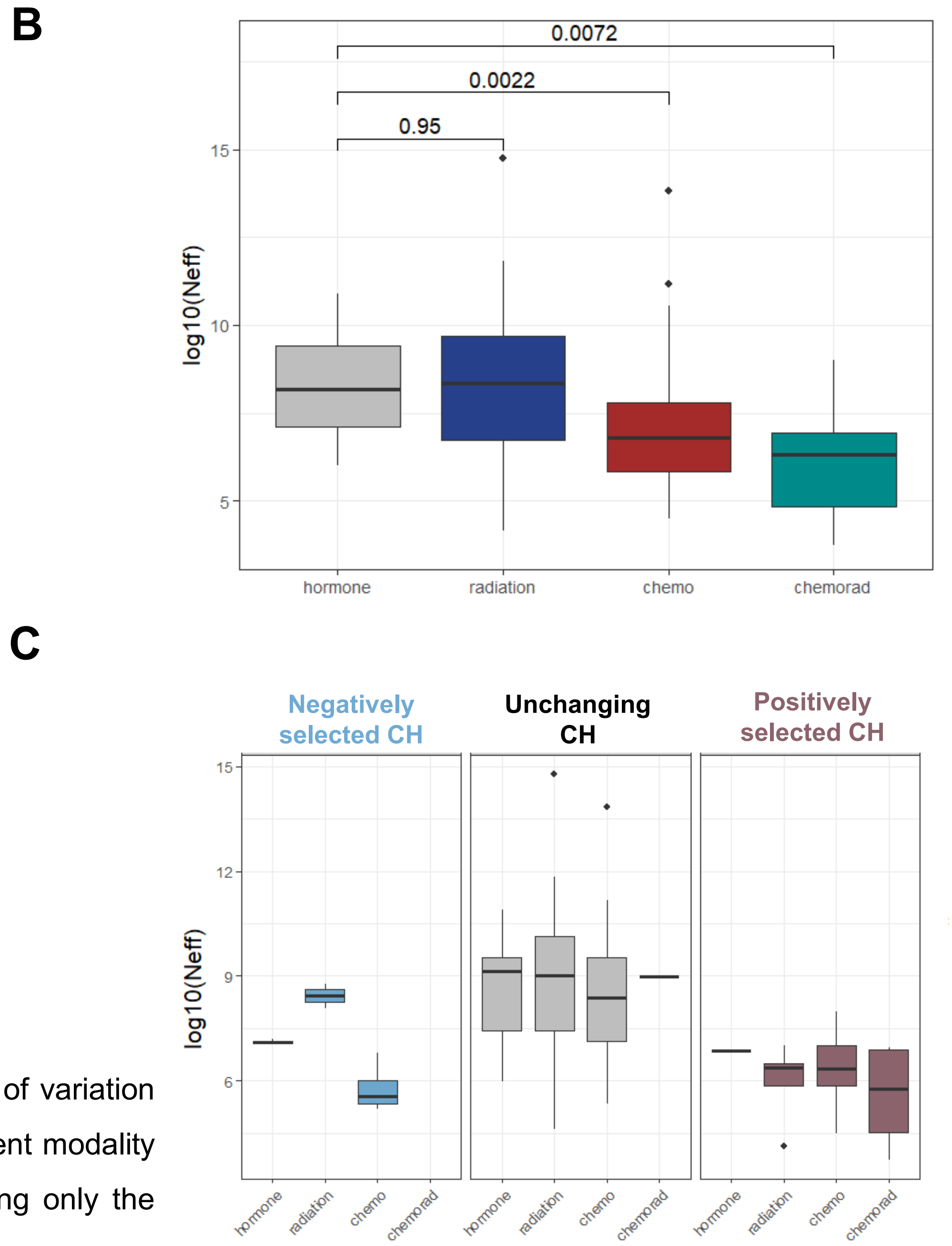

FIGURE S10. Sensitivity analyses for allelic population size (N_{eff}) assumptions. A) Coefficient of variation for N_{eff} calculated across the cohort at different sequencing depths. B) N_{eff} stratified by treatment modality and, C) dynamic selection groups; with the assumption of clonal dependency and considering only the largest clone in each sample.
